# MUSASHI-Mediated Expression of JMJD3, a H3K27me3 Demethylase, Is Involved in Foamy Macrophage Generation during Mycobacterial Infection

**DOI:** 10.1371/journal.ppat.1005814

**Published:** 2016-08-17

**Authors:** Sahana Holla, Praveen Prakhar, Vikas Singh, Anupama Karnam, Tanushree Mukherjee, Kasturi Mahadik, Pankti Parikh, Amit Singh, R. S. Rajmani, Subbaraya G. Ramachandra, Kithiganahalli Narayanaswamy Balaji

**Affiliations:** 1 Department of Microbiology and Cell Biology, Indian Institute of Science, Bangalore, Karnataka, India; 2 Department of Microbiology and Cell Biology, Centre for Infectious Disease Research, Indian Institute of Science, Bangalore, Karnataka, India; 3 Central Animal Facility, Indian Institute of Science, Bangalore, Karnataka, India; Harvard School of Public Health, UNITED STATES

## Abstract

Foamy macrophages (FM)s harbor lipid bodies that not only assist mycobacterial persistence within the granulomas but also are sites for intracellular signaling and inflammatory mediators which are essential for mycobacterial pathogenesis. However, molecular mechanisms that regulate intracellular lipid accumulation in FMs during mycobacterial infection are not clear. Here, we report for the first time that jumonji domain containing protein (JMJD)3, a demethylase of the repressive H3K27me3 mark, orchestrates the expression of *M*. *tuberculosis* H37Rv-, MDR-JAL2287-, H37Ra- and *M*. *bovis* BCG-induced genes essential for FM generation in a TLR2-dependent manner. Further, NOTCH1-responsive RNA-binding protein MUSASHI (MSI), targets a transcriptional repressor of JMJD3, Msx2-interacting nuclear target protein, to positively regulate infection-induced JMJD3 expression, FM generation and M2 phenotype. Investigations in *in vivo* murine models further substantiated these observations. Together, our study has attributed novel roles for JMJD3 and its regulators during mycobacterial infection that assist FM generation and fine-tune associated host immunity.

## Author Summary

Foamy macrophages (FMs) not only provide a suitable survival niche for the mycobacteria in the granuloma but also are reservoirs for several inflammatory mediators that regulate mycobacterial pathogenesis. Hence, understanding the mechanisms that regulate infection-induced FM generation assumes importance. In this investigation, we present empirical evidence to support the role of host epigenetic mechanisms in generating FMs and thus facilitating mycobacterial persistence *in vivo*. We show that the signaling pathways that mediate mycobacteria-induced expression of JMJD3, a demethylase of the facultative repression mark, regulate the genes assisting in FM generation. Importantly, the identified pathway could largely contribute to the evasive responses during mycobacterial infection and suppression of such pathways during infection could confer stronger immunity. Together, these regulators could be potential candidates for host-directed therapies against mycobacterial infection.

## Introduction

Macrophages recruit to the site/s of mycobacterial infection and differentiate to several cell types including the lipid droplet loaded foamy macrophages (FMs) [1]. The characteristic lipid body (LB) generation in the FMs provides a suitable survival niche for the mycobacteria in the granuloma [2]. It is known that FMs not only provide the nutrient source for the internalized mycobacteria, but also generate an environment conducive for the bacilli to attain the non-replicating persistence state [3]. More importantly, FMs are the reservoirs for several inflammatory mediators such as arachidonic acid and the enzymes that catalyze the conversion of arachidonic acid to immunomodulatory eicosanoids like cyclooxygenase-2, lipoxygenases-5 and -15 and LTC4-synthase [1]. Additionally, LBs also regulate lipid metabolism, membrane trafficking and intracellular signaling [4]. Hence, understanding the mechanisms that regulate mycobacterial infection-induced FM generation assumes importance.

A complex co-ordinated mechanism of lipid influx, metabolism, storage and mobilization constitute the FM generation [4]. These processes are carried out by a dedicated set of diverse genes. The genes that assist in lipid biosynthesis like acyl-CoA synthetase long-chain family member-1 (*Acsl1*, lipid biosynthesis), adipose differentiation-related protein (*Adrp/Plin2*, lipid droplet synthesis) and sphingolipid activator proteins (*Psap*/SapC, its product maintains the turnover of lipids in membranes) are known to be upregulated during FM formation [1, 5]. Membrane proteins like fatty acid translocase (*Fat*/CD36), macrophage scavenger receptor-1 (*Msr1*/CD204) and macrophage receptor with collagenous domain (*Marco*) were essential for uptake of low-density lipoproteins into the macrophages, a characteristic evidence for FM generation [3]. The low-density lipoproteins inside the FMs are metabolized to triacylglycerides, phospholipids and cholesterol. The esterified cholesterol either gets sequestered to the LBs or is effluxed via the ATP-binding cassette (ABC) transporters, *Abca1* and *Abcg1* [3]. Hence, deficiency or downregulation of the ABC transporters favour FM generation [6]. Importantly, fine-regulation of the above mentioned genes would orchestrate the FM phenotype and functions during mycobacterial pathogenesis.

In this context, regulatory mechanisms governing such pathogen-specific spatio-temporal inflammatory responses would involve reversible, instantaneous but specific action like the ones mediated by epigenetic regulators [7]. Of the various epigenetic mechanisms, histone modifications play vital roles in regulating the gene expression [8]. Interestingly, many histone marks including Histone H3 lysine 27 trimethylation (H3K27me3) have been implicated in inflammation and pathogenesis [9]. It is well established that H3K27me3 brings about the silencing of genes [10]. In general, trimethylation of H3K27 is catalyzed by EZH2, which associates with SUZ12, EED and RbAp48 to form the polycomb-repressive complex 2 (PRC2) and jumonji domain containing protein (JMJD)3 is a known H3K27me3 demethylase [10]. Importantly, PRC2 complex is a potent regulator of several signaling pathways like NOTCH1, WNT and sonic hedgehog signaling [11] which have been reported to be activated during mycobacterial infection to direct the immune responses and determine the cell-fate [12–15]. Additionally, reports have implicated the role for JMJD3 in regulating inflammation and TLR responses [10, 16, 17] including generation of M2 phenotype [18] and foamy characteristics of macrophages during atherosclerosis [19]. Of note, M2 macrophages function to exacerbate mycobacterial pathogenesis [15, 20–22] and FM molecular markers such CD36, MSR1, lipoxygenases 5/15 etc constitute M2 macrophages [23, 24]. In this perspective, the role for H3K27 methylation by PRC2 complex and its demethylase, JMJD3 during mycobacteria-responsive FM generation was explored.

Infection of macrophages with *M*. *tuberculosis* H37Rv (represented as H37Rv), multi-drug resistant strain MDR-JAL2287, *M*. *tuberculosis* H37Ra (represented as H37Ra) or *M*. *bovis* BCG (represented as BCG), but not *M*. *smegmatis*, displayed JMJD3-dependent LB formation. Supporting this observation, the genes involved in lipid biosynthesis (*Acsl1*, *Adrp*, *Psap*) and uptake (*Fat/*CD36) and *Msr1*) were significantly upregulated with mycobacterial infection of macrophages in a JMJD3- and TLR2-dependent manner. Deciphering the mechanism of JMJD3 expression, we found an evolutionarily conserved RNA-binding protein MUSASHI (MSI), which was NOTCH1-responsive, to target a transcriptional repressor of JMJD3, Msx2-interacting nuclear target protein (MINT/*Spen*) and thus assist in FM generation during mycobacterial infection. Immunohistochemistry (IHC) and immunofluorescence (IF) experiments utilizing H37Rv-infected lungs and *in vivo* murine BCG-induced granuloma model substantiated these observations. MSI-JMJD3 axis was found to regulate M2 phenotypic responses in the FMs during mycobacterial infection. Thus, the current investigation has identified roles for JMJD3 and associated epigenetic regulators to shape the immune responses during mycobacterial pathogenesis.

## Results

### TLR2 signaling mediates JMJD3-dependent FM formation during mycobacterial infection

**Fig 1:**
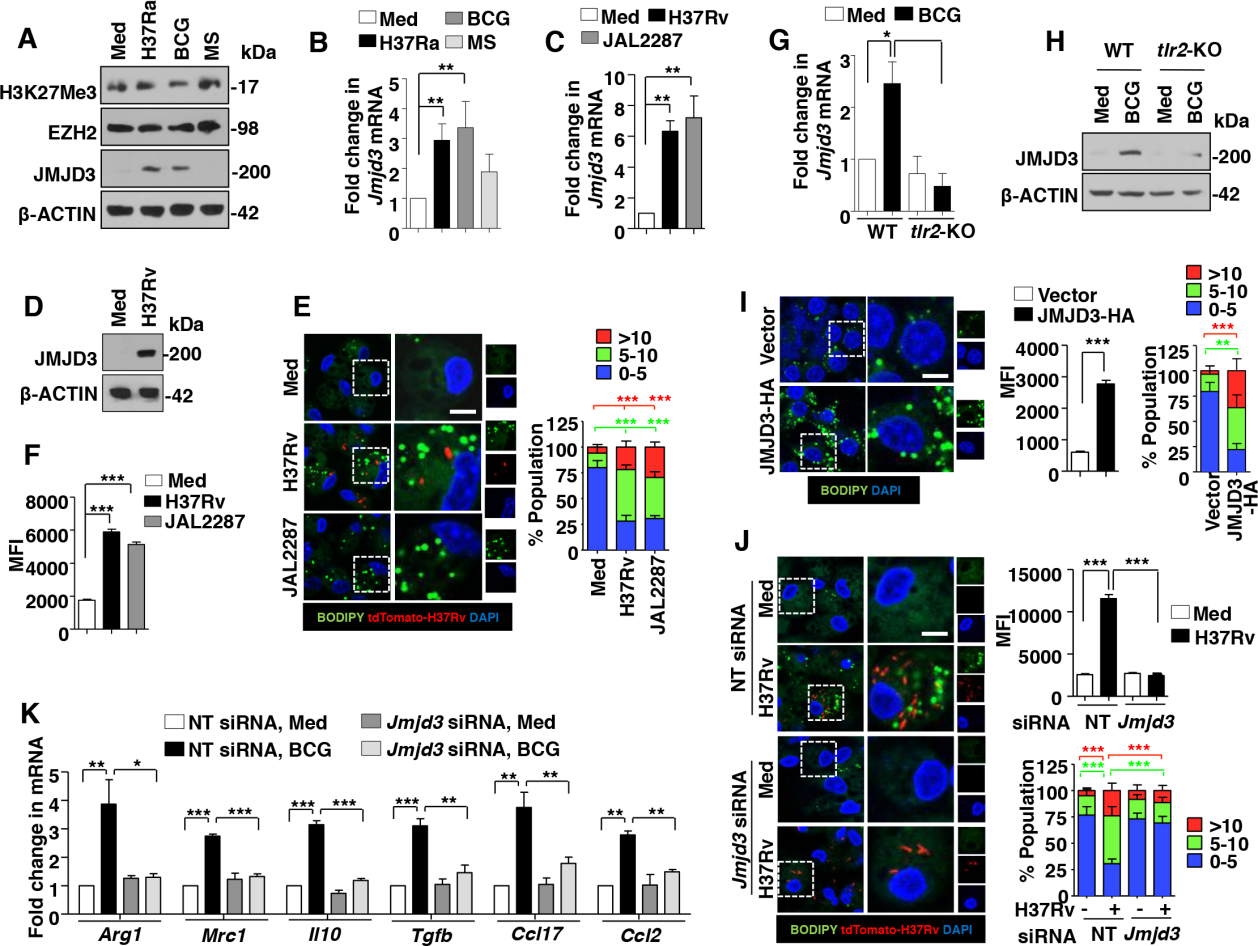
TLR2-responsive JMJD3 regulates mycobacteria-induced FM formation and immune responses. (A and B) Mouse peritoneal macrophages were infected with the indicated bacteria (H37Ra: *M*. *tuberculosis* H37Ra; BCG: *M*. *bovis* BCG; MS: *M*. *smegmatis*) for 6 h. Immunoblot analysis for H3K27me3, EZH2 and JMJD3 (A) and quantitative real-time RT-PCR for *Jmjd3* expression (B). (C and D) Transcript (C) and protein (D) levels of JMJD3 in H37Rv- and MDR-JAL2287-infected peritoneal macrophages. (E and F) IF imaging of BODIPY-stained lipid droplets in peritoneal macrophages infected for 48 h with H37Rv or MDR-JAL2287 (E, left panel). DNA was stained with DAPI. Frequency of FMs was calculated by counting the population of cells expressing 0–5, 5–10 or >10 lipid bodies (n = 250–300) and plotted as a bar graph (E, right panel). Based on the IF images, MFIs were calculated (n = 100, each treatment) and plotted (F). (G and H) Peritoneal macrophages from WT or *tlr2*-null mice were infected with BCG for 6 h and quantitative real-time RT-PCR (G) and immunoblotting (H) were performed to assess the expression levels of JMJD3. (I and J) RAW 264.7 cells were transiently transfected with JMJD3-HA (I) and peritoneal macrophages transfected with NT or *Jmjd3* siRNA were infected with H37Rv expressing the red fluorescent protein tdTomato for 48 h (J). Representative IF images of macrophages with BODIPY-stained lipid droplets (left panels). MFIs (middle panel in I and right top panel in J) and frequency of FMs (right panels) were calculated as mentioned in panels E and F. (K) RAW 264.7 macrophages transiently transfected NT or *Jmjd3* siRNA were infected with BCG for 12 h. Quantitative real-time RT-PCR for the indicated M2 markers. All data represents the mean ± SEM for at least 3 independent experiments, *P < 0.05, **P < 0.005, ***P < 0.0005 (one-way ANOVA followed by Tukey’s multiple-comparisons test except for two-tailed paired Student’s *t*-test in I) and all blots are representative of at least 3 independent experiments. Med, medium; WT, wild-type; KO, knockout; NT, non-targeting; MFI, mean fluorescence intensity. Bar, 5 μm; Original magnifications 63X.

FMs are the integral components of granulomas during mycobacterial pathogenesis [2]. However, mechanisms that regulate intracellular lipid accumulation in the FMs during the course of mycobacterial infection require extensive investigation. To begin with, the ability of different mycobacterial species to induce FMs was analyzed. H37Ra- and BCG-infected RAW 264.7 macrophages, unlike *M*. *smegmatis*, stained positive for LB formation as assessed by Oil Red O (ORO) staining and measurement of the extracted ORO (S1A Fig). With reports suggesting a crucial role for TLR2 signaling during FM generation [25] and TLR2 signaling being a major mediator of mycobacterial pathogenesis [26], analysis of the contribution of TLR2 during mycobacteria-mediated FM formation was undertaken. In this regard, RAW 264.7 macrophages expressing the dominant negative form of TLR2 failed to generate FMs on H37Ra and BCG infection (S1A Fig). Additionally, lipid bodies in primary mouse macrophages were labeled with fluorescent dye BODIPY 493/503 to assess the frequency and mean fluorescence intensities (MFIs) of FMs. In line with the ORO results, H37Ra and BCG showed increased frequency and MFIs of BODIPY-stained macrophages in WT mice when compared to that in *tlr2*-null mice (S1B Fig). For the reasons mentioned earlier in the introduction and to understand the mechanisms governing such mycobacteria-TLR2-specific responses, we sought to uncover the role for epigenetic regulation, if any, of the coordinated process of FM generation. Of note, H3K27me3 is a histone mark widely implicated during inflammation and pathogenesis [27]. Hence, H3K27-associated methylase EZH2 and demethylase JMJD3 were analyzed to explore the epigenetic regulation of TLR2-dependend FM induction during mycobacterial infections. Primary macrophages infected with H37Ra or BCG, but not *M*. *smegmatis*, were found to display elevated expression of JMJD3 at both RNA and protein levels (Fig 1A and 1B). However, no significant global change in the levels of H3K27me3 and EZH2 were observed (Fig 1A). Interestingly, peritoneal macrophages infected with virulent strains of *M*. *tuberculosis* like H37Rv and MDR-JAL2287 induced a robust expression of JMJD3 (Fig 1C and 1D). Like avirulent strain of mycobacteria, H37Rv and MDR-JAL2287 showed increased frequency and MFIs of BODIPY-stained macrophages (Fig 1E and 1F) and ORO staining (S1C Fig), indicative of significant FM generation. Role for TLR2 in mediating the mycobacteria-induced JMJD3 expression was verified in macrophages obtained from *tlr2*-null mice (Fig 1G and 1H). To establish the physiological role for JMJD3 during LB generation, overexpression and knockdown experiments of JMJD3 were performed. While RAW 264.7 macrophages overexpressing JMJD3 showed significant induction of the FMs (Fig 1I and S1D and S1E Fig), macrophages depleted of JMJD3 using specific siRNAs displayed compromised ability to generate FMs on mycobacterial infection of H37Rv and BCG (Fig 1J and S1F and S1G Fig). As mentioned earlier, FMs house several inflammatory mediators that contribute to the M2 phenotypic and functional properties of FMs in different disease contexts [23, 24]. We thus assessed the role for JMJD3 in orchestrating the immune responses exhibited by FMs. Interestingly, while JMJD3 was found to negatively regulate few of the mycobacteria-responsive M1 markers of macrophages *viz*., *Il12*, *Il1b* and *Cxcl2* (S2A Fig), the expression of M2 markers like *Arg1*, *Mrc1*, *Il10*, *Tgfb*, *Ccl17* and *Ccl2* on infection were JMJD3-dependent (Fig 1K).

**Fig 2:**
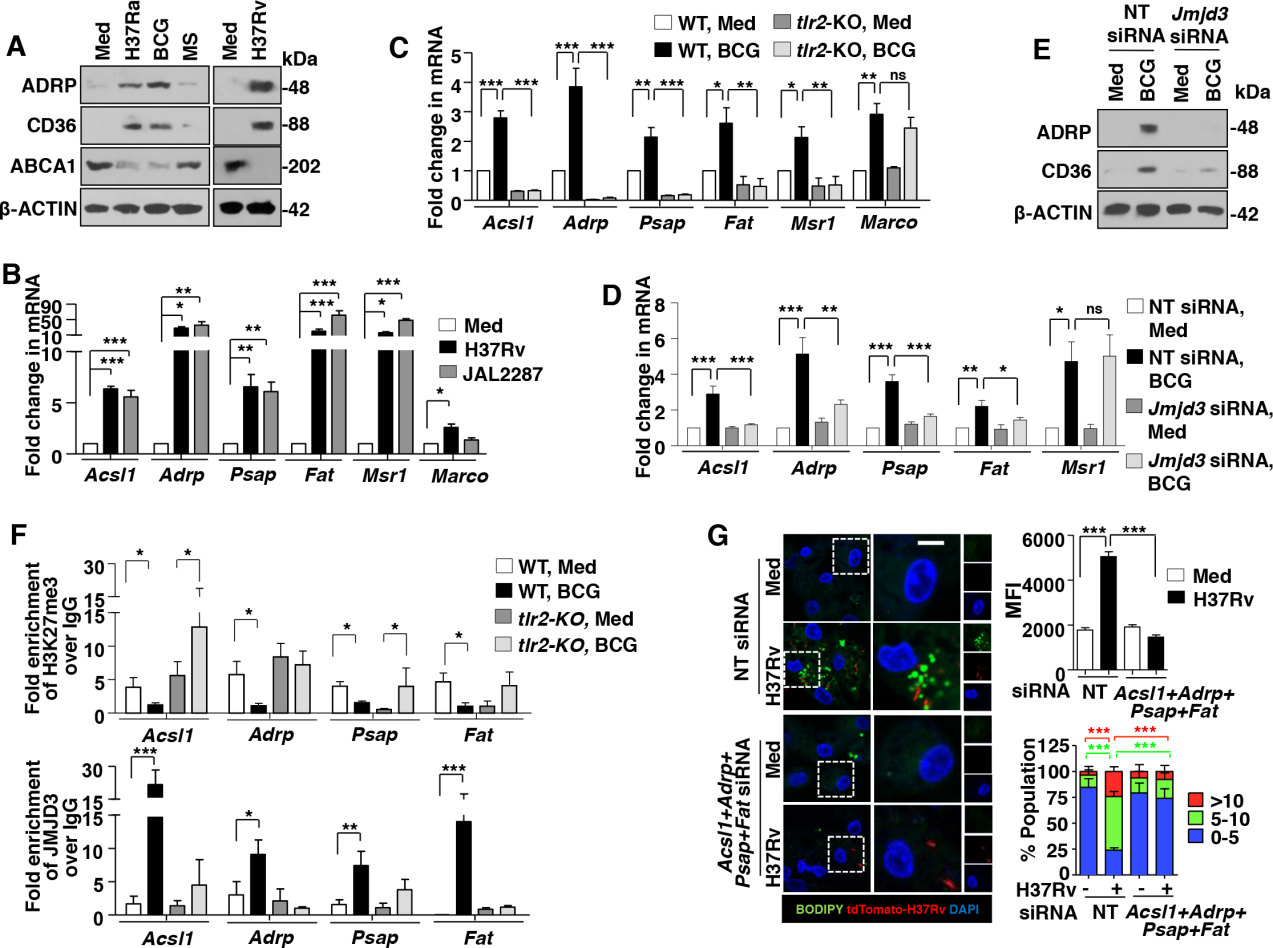
JMJD3 epigenetically regulates the expression of genes required for mycobacteria-induced FMs. (A and B) Protein (A) and transcript (B) analysis of the selected genes involved during FM formation in peritoneal macrophages infected with the indicated bacteria for 12 h. (C) Peritoneal macrophages from WT or *tlr2*-null mice were infected with BCG for 12 h and quantitative real-time RT-PCR for the selected FM genes. (D and E) Murine RAW 264.7 cells were transiently transfected with NT or *Jmjd3* siRNA and infected with BCG for 12 h. Quantitative real-time RT-PCR (D) and immunoblotting (E) for the selected FM genes. (F) Peritoneal macrophages from WT or *tlr2*-null mice were infected with BCG for 12 h. H3K27me3 modification and JMJD3 recruitment at promoters of *Acsl1*, *Adrp*, *Psap* and *Fat* were evaluated by ChIP. (G) Peritoneal macrophages transfected with NT or *Acsl1+Adrp+Psap+Fat* siRNA were infected with H37Rv expressing the red fluorescent protein tdTomato for 48 h. Representative IF images of macrophages with BODIPY-stained lipid droplets (left panel). Based on the IF images, MFIs were calculated (n = 100, each treatment) and plotted (top right panel). Frequency of FMs was calculated by counting the population of cells expressing 0–5, 5–10 or >10 lipid bodies (n = 250–300) and plotted as a bar graph (bottom right panel). All data represents the mean ± SEM for at least 3 independent experiments, ns = not significant, *P < 0.05, **P < 0.005, ***P < 0.0005 (one-way ANOVA followed by Tukey’s multiple-comparisons test) and all blots are representative of 3 independent experiments. Med, medium; NT, non-targeting; WT, wild-type; KO, knockout; MFI, mean fluorescence intensity. Bar, 5 μm; Original magnifications 63X.

Several genes co-ordinate the lipid biosynthesis, uptake and accumulation processes during FM formation [5]. Expression analysis of few of the genes suggested that H37Ra and BCG, but not *M*. *smegmatis*, induced the positive regulators (*Acsl1*, *Adrp*, *Psap*, *Fat*, *Msr1* and *Marco*) and downregulated or did not modify the negative regulators (*Abca1* and *Abcg1*) of FM generation (S2B Fig and Fig 2A). In accordance with the previous results utilizing virulent mycobacterial strains, H37Rv and MDR-JAL2287 expressed the FM genes (Fig 2B). Further, mycobacteria-responsive expression of *Acsl1*, *Adrp*, *Psap*, *Fat* and *Msr1* was found to be TLR2-dependent (Fig 2C). Since induced expression of JMJD3 was found essential to render the FM phenotype during mycobacterial infection (Fig 1J), contribution of JMJD3 in regulating the identified set of genes was assessed. Corroborating the previous observation, RAW 264.7 macrophages expressing *Jmjd3*-specific siRNAs failed to express *Acsl1*, *Adrp*, *Psap* and *Fat* on BCG infection (Fig 2D and 2E). To further validate the epigenetic regulation of the identified genes, ChIP experiments were performed. BCG infection of primary macrophages showed TLR2-dependency for decreased H3K27me3 methylations on the promoters of *Acsl1*, *Adrp*, *Psap* and *Fat* genes marking the active transcription (Fig 2F). Importantly, corresponding recruitment of JMJD3 to the identified promoters was found. While BCG infection of wild-type macrophage showed significant recruitment of JMJD3 to the promoters of *Acsl1*, *Adrp*, *Psap* and *Fat* genes, macrophages from *tlr2*-null mice did not exhibit similar results (Fig 2F). Possibility of direct regulation of the M2 genes by JMJD3 was ruled out as ChIP results suggested that mycobacterial infection does not modulate H3K27me3 modification or recruitment of JMJD3 to the promoters of M2 genes in macrophages (S2C Fig). Additionally, BODIPY and transcript analysis in macrophages transfected with specific siRNAs to *Acsl1*, *Adrp*, *Psap* and *Fat* genes underscored the role for these genes in regulating FM generation (Fig 2G and S2D Fig) and M2 gene expression (S2E Fig). Together, this suggests that mycobacteria-induced TLR2 signaling directs the JMJD3-dependent expression of genes required for LB formation, FM generation and concomitant M2 phenotypic responses.

### TLR2-dependent FM formation and JMJD3 expression in *in vivo* murine models

**Fig 3:**
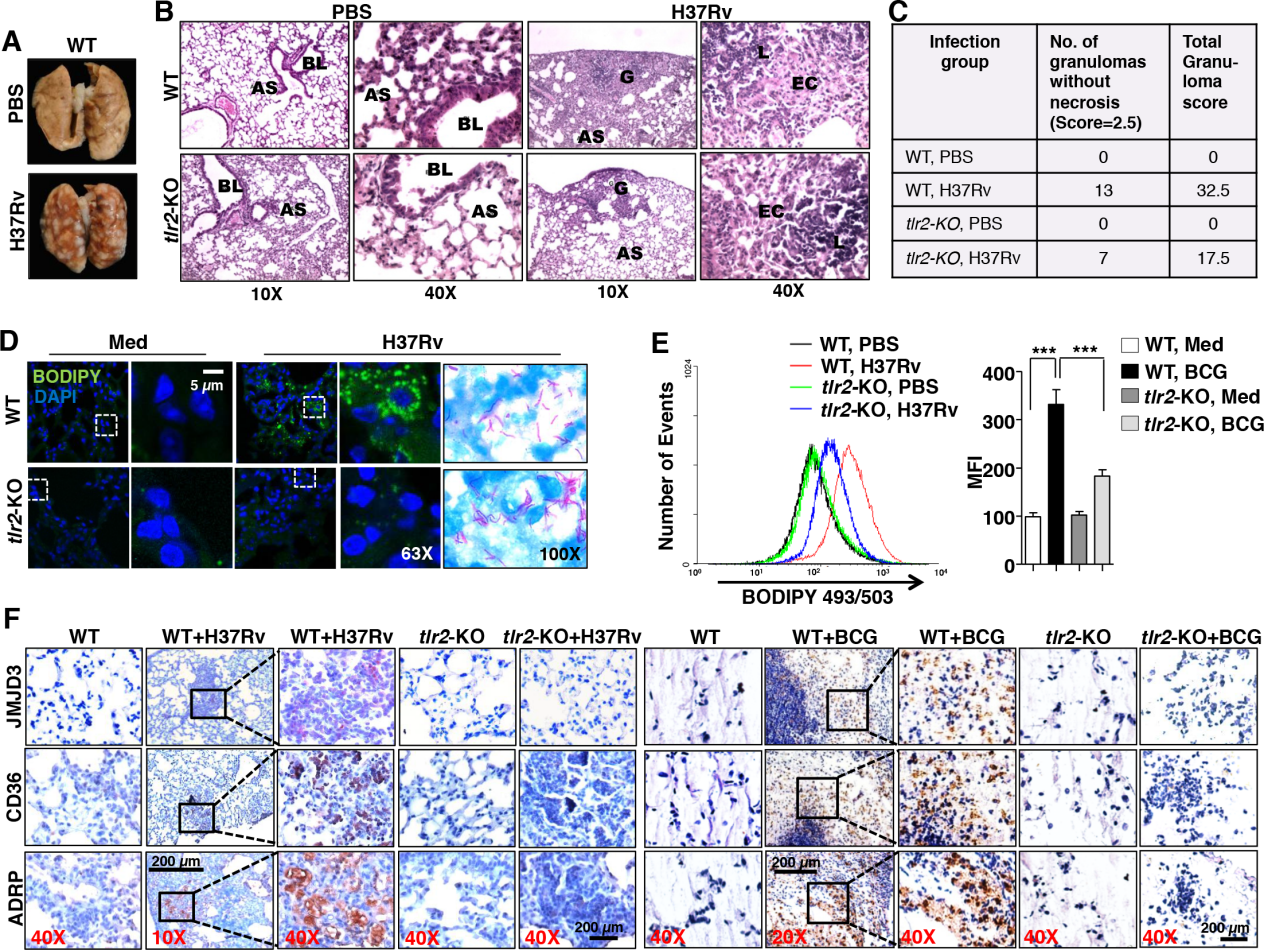
*In vivo* murine granuloma models display TLR2-dependent FM generation and expression of associated genes. (A-E) WT or *tlr2*-null mice were infected by aerosol inhalation of 500 CFUs of H37Rv (n = 6 in each group, two independent experiment). Pulmonary pathology of the infected lungs (A). Representative images of hematoxylin and eosin staining of formaldehyde-fixed, paraffin-embedded lung tissue sections are shown (B). Lungs from PBS treated mice (left two panels): Low power (10X) photomicrograph of a lung showing normal pulmonary parenchyma and high power (40X) of same section showing part of normal bronchial wall and alveoli are represented. BL = Bronchial Lumen, AS = Alveolar Space. Lungs from H37Rv treated mice (right two panels): Low power (10X) photomicrograph of a lung showing a granuloma on the periphery and high power (40X) photomicrograph showing the granuloma formed of epithelioid cells and lymphocytes are represented. No necrosis was observed in granulomas from both WT and *tlr2*-null mice lungs. G = Granuloma, AS = Alveolar Space, EC = Epithelioid cells, L = Lymphocytes (B). Tabulation of granuloma scores from histopathology results (C). IF imaging of BODIPY-stained lipid droplets in the cryosections of the lungs (D). Formaldehyde-fixed, paraffin-embedded lung sections from H37Rv-infected WT and *tlr2*-KO mice were stained for acid-fast bacteria by Ziehl-Neelsen method (D, right most panels). Flow cytometry of BODIPY-stained lung cells (E). Histogram plot for BODIPY 493/503 fluorescence (left panel) and MFIs (right panel) of the same are plotted. Data represents the mean ± SEM (n = 4), ***P < 0.0005 (one-way ANOVA followed by Tukey’s multiple-comparisons test). (F) IHC on formaldehyde-fixed, paraffin-embedded lung (left 5 panels)/ granuloma (right 5 panels) sections from WT and *tlr2*-KO mice was performed to assess the *in vivo* expression of JMJD3, ADRP and CD36. Representative images are shown here (n = 6). WT, wild-type; KO, knockout; MFI, mean fluorescence intensity. Original magnifications and scale are indicated on the images.

To bring the *in vivo* relevance of the identified mechanism of FM generation, we utilized two murine models. Lungs were isolated from WT and *tlr2*-null mice after aerosol infection with H37Rv. Characteristic lesions were observed in larger numbers on the pleura of infected WT mice as compared to that in infected *tlr2*-null mice (S3A Fig and Fig 3A). Analysis of lung tissue sections revealed characteristic granulomas with epithelioid cells and lymphocytes in the H37Rv-infected mice. However, no necrosis was observed. While 5–8 such granulomas were observed in the lungs of the WT mice, *tlr2-KO* mice were found to have 2–3 granulomas in their lungs. Importantly, the granuloma score was significantly reduced from 32.5 in the WT to 17.5 in the *tlr2-KO* mice (Fig 3B and 3C), indicating the TLR2 dependency of mycobacteria-induced granulomas. Further, BODIPY analysis of the lungs by IF (Fig 3D) and FACS (Fig 3E) and ORO staining (S3B Fig) validated the TLR2-dependent FM generation in mice during H37Rv infection. In an alternate study, a previously well known *in vivo* murine granuloma model [5, 28] was established with BCG infection. The excised granulomas were analyzed for the characteristic hallmarks of a granuloma such as cellular architecture, peripheral accumulation of the lymphocytes and different classes of macrophages constituting the center (S3C Fig). After authenticating the obtained granulomas, ORO staining of the sections were performed. BCG-induced granulomas from wild-type mice displayed increased occurrence of FMs in the tissues when compared to that from *tlr2*-null mice (S3D Fig). Importantly, results from IHC (Fig 3F) and IF (S3E Fig) experiments suggested that while H37Rv- and BCG-induced granulomas from wild-type mice express elevated levels of JMJD3, ADRP and CD36, expression of these genes in granulomas from *tlr2*-null mice was significantly abrogated.

### Mycobacteria downregulate MINT/*Spen* to facilitate JMJD3 expression

**Fig 4:**
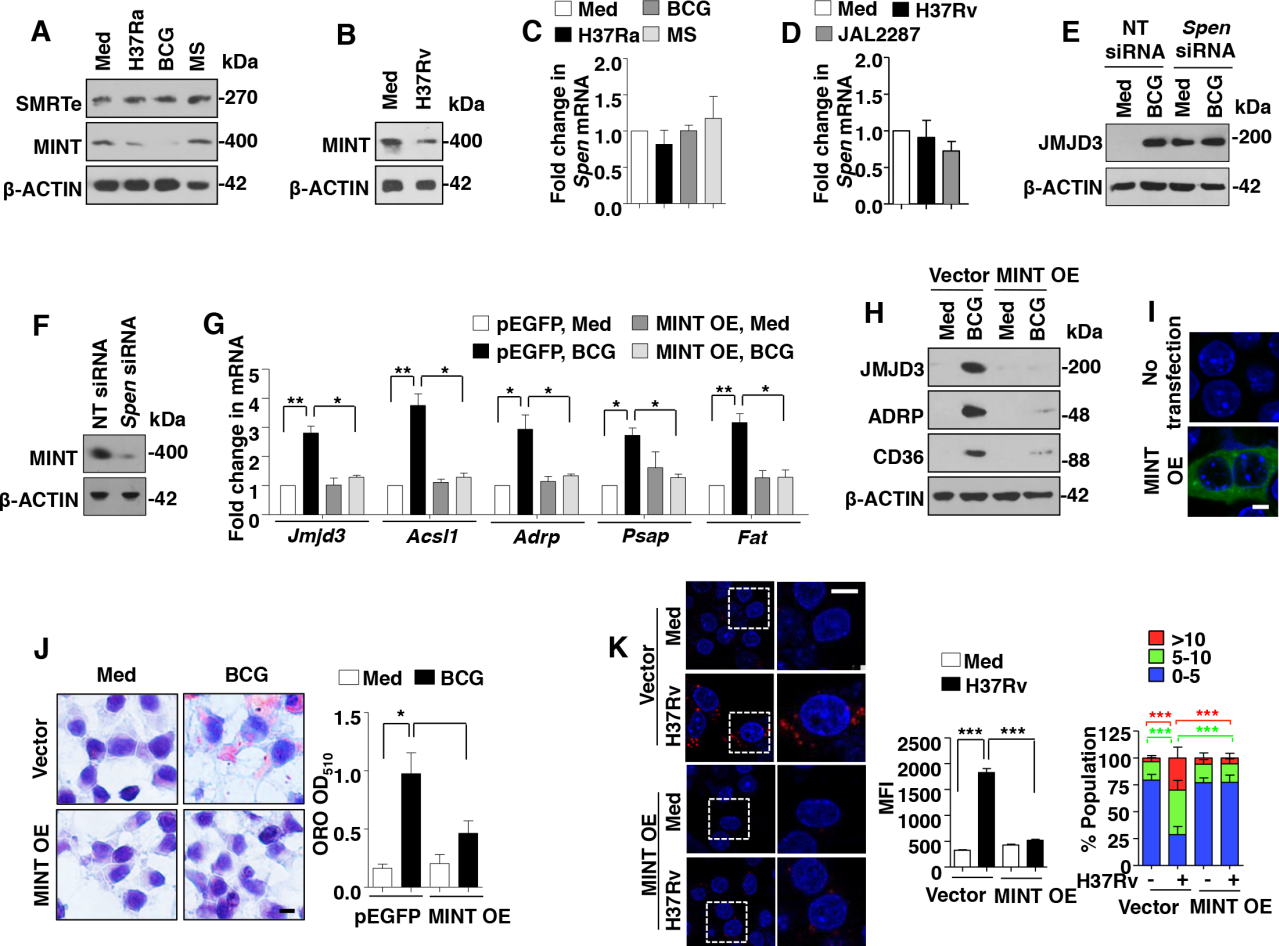
MINT/*Spen*, a negative regulator of JMJD3, is downregulated during mycobacterial infection. (A-D) Mouse peritoneal macrophages were infected with the indicated bacteria for 6 h. Immunoblot analysis for SMRTe and MINT (A and B) and quantitative real-time RT-PCR for *Spen* expression (C and D). (E and F) Murine RAW 264.7 cells were transiently transfected with NT or *Spen* siRNA and infected with BCG for 12 h. Immunoblotting analysis of the lysates for expression of JMJD3 (E) and MINT (F). (G-J) Transient transfection of RAW 264.7 cells with MINT OE construct in presence of BCG infection for 12 h (G-I) or 48 h (J) was performed. Transcript (G) and protein (H) level expression of JMJD3 and other selected genes were assayed by quantitative real-time RT-PCR and immunoblotting respectively. Validation of MINT OE (MINT-EGFP) construct (I). Representative images of cells stained with ORO (J, left panel) and extracted ORO was measured at OD_510_ (J, right panel). (K) RAW 264.7 cells transiently transfected with MINT OE were infected with H37Rv for 48 h. Representative IF images of macrophages with LipidTOX Red-stained lipid droplets (left panel). Based on the IF images, MFIs were calculated (n = 100, each treatment) and plotted (top right panel). Frequency of FMs was calculated by counting the population of cells expressing 0–5, 5–10 or >10 lipid bodies (n = 250–300) and plotted as a bar graph (bottom right panel). All data represents the mean ± SEM for at least 3 independent experiments, *P < 0.05, **P < 0.005, ***P < 0.0005 (one-way ANOVA followed by Tukey’s multiple-comparisons test) and all blots are representative of 3 independent experiments. Med, medium; NT, non-targeting; OE, overexpression; MFI, mean fluorescence intensity. Bar, 5 μm; Original magnifications 63X in I and K and 100X in J.

After establishing the downstream effector functions of JMJD3, the possible mechanisms by which mycobacteria induces JMJD3 expression were explored. In this context, silencing mediator for RARs and thyroid receptors-extended (SMRTe/NCoR2) is a known repressor of JMJD3 expression in neuronal cells [29]. Importantly, SMRTe complexes with MINT/*Spen*, HDAC1 and HDAC2 to bring about the repression of the target genes [30]. Hence, the role for SMRTe and MINT/*Spen* to regulate JMJD3 expression was assessed. As shown in Fig 4A and 4B, while no significant change in the expression of SMRTe was observed, expression of MINT protein was significantly downregulated on H37Rv, H37Ra and BCG infection in primary macrophages. This observation corresponds to the induced expression of JMJD3 in these conditions (Fig 1A–1D). However, levels of *Spen* transcripts did not alter with the infection (Fig 4C and 4D). Interestingly, we found that inhibition of MINT by specific siRNA results in significant increase in JMJD3 expression, even in the absence of infection (Fig 4E and 4F). To further establish the role for MINT in negatively regulating JMJD3 responses, MINT was overexpressed in RAW 264.7 macrophages. Ectopic expression of MINT not only suppressed the ability of mycobacteria (both BCG and H37Rv) to induce JMJD3 and the downstream genes responsible for FM generation (Fig 4G, 4H and 4I), but also compromised the ability of BCG to form FMs (Fig 4J and 4K). Thus, mycobacteria subdue the expression of MINT to elevate JMJD3 expression and mediate FM formation.

### MSI targets MINT to regulate JMJD3 expression and FM generation

**Fig 5:**
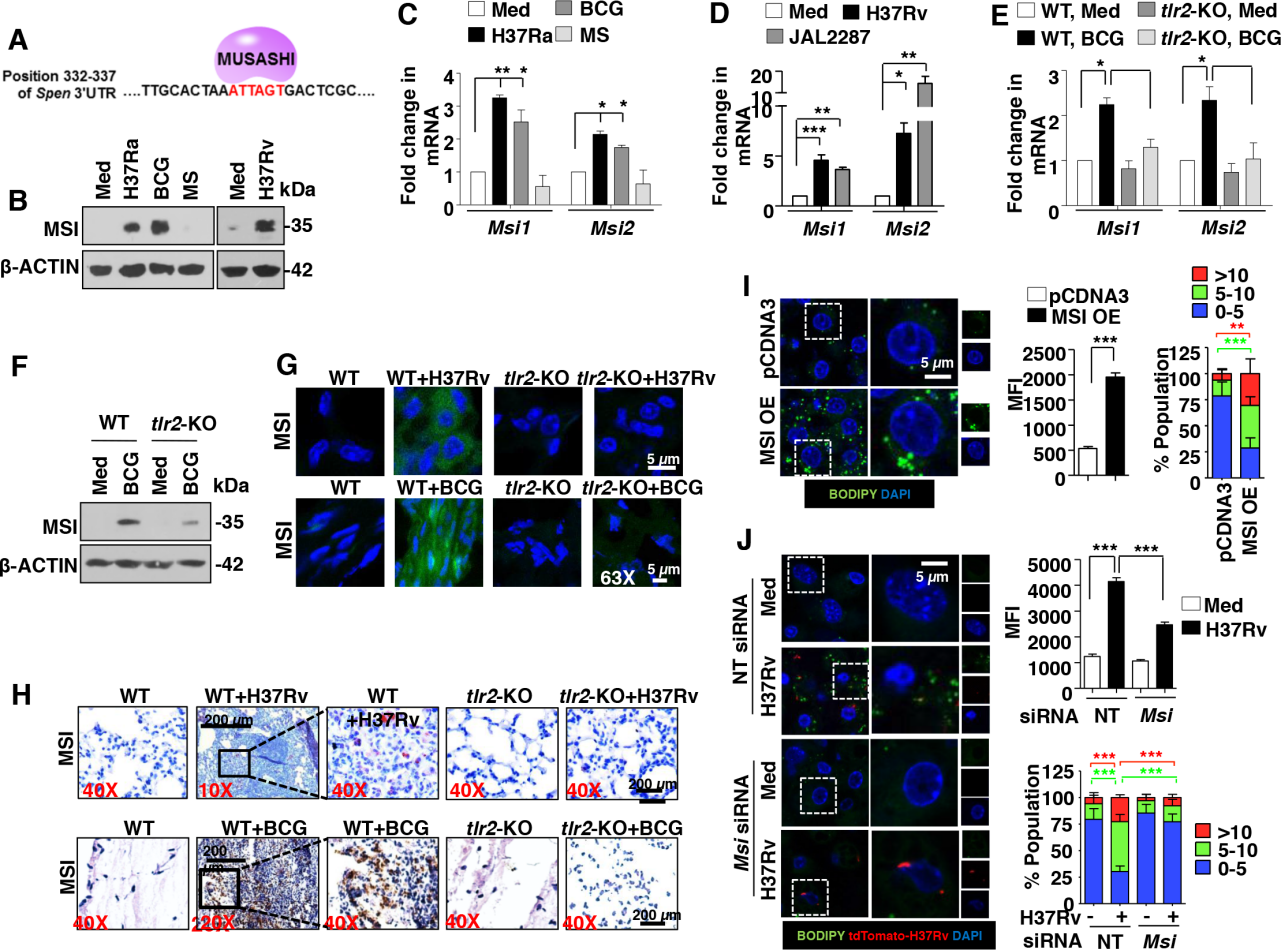
Mycobacteria-induced expression of MSI is requisite for FM formation. (A) MSI-binding site, ATTAGT spanning the 332–337 residues of the *Spen* 3’UTR. (B-D) Mouse peritoneal macrophages were infected with the indicated bacteria for 6 h. Immunoblot analysis MSI (B) and quantitative real-time RT-PCR for *Msi* isoforms expression (C and D). (E and F) Transcript (E) and protein (F) levels of MSI in 6 h BCG-infected peritoneal macrophages from WT or *tlr2*-KO mice. (G and H) *In vivo* expression of MSI in the H37Rv-infected lung sections (upper panels) and BCG-induced granulomas (lower panels) by IF (G) and IHC (H) studies. (I and J) RAW 264.7 cells were transiently transfected with MSI1 OE (I) or peritoneal macrophages transfected with NT or *Msi* siRNA were infected with H37Rv expressing the red fluorescent protein tdTomato for 48 h. Representative IF images of macrophages with BODIPY-stained lipid droplets (left panels). Based on the IF images, MFIs were calculated (n = 100, each treatment) and plotted (middle panels). Frequency of FMs was calculated by counting the population of cells expressing 0–5, 5–10 or >10 lipid bodies (n = 250–300) and plotted as a bar graph (right panels). All data represents the mean ± SEM for at least 3 independent experiments, *P < 0.05, **P < 0.005, ***P < 0.0005 (one-way ANOVA followed by Tukey’s multiple-comparisons test except for two-tailed paired Student’s *t*-test in I) and all blots are representative of 3 independent experiments. Med, medium; WT, wild-type; KO, knockout; OE, overexpression; NT, non-targeting; MFI, mean fluorescence intensity. Original magnifications and scale for G and H are indicated on the images. Original magnifications 63X and Bar, 5 μm in I and J.

Interestingly, as shown, mycobacteria-mediated inhibition of MINT/*Spen* was observed at the protein but not at transcript levels (Fig 4A–4D). This underscores a regulation-mediated by post-transcriptional modifications. One such known regulatory mechanism is exhibited by a RNA binding protein, MSI [31]. MSI isoforms MSI1 and MSI2, bind to the 3’UTR of the target mRNA to block its translation [32]. In the current context, *Spen* 3’UTR was analyzed for the binding site of MSI, (G/A)U_n_AGU (n = 2–3) [32]. Importantly, a binding site ATTAGT spanning the 332–337 residues of the *Spen* 3’UTR was identified (Fig 5A). Thus, it was hypothesized that mycobacteria may regulate MINT via MSI activity.

**Fig 6:**
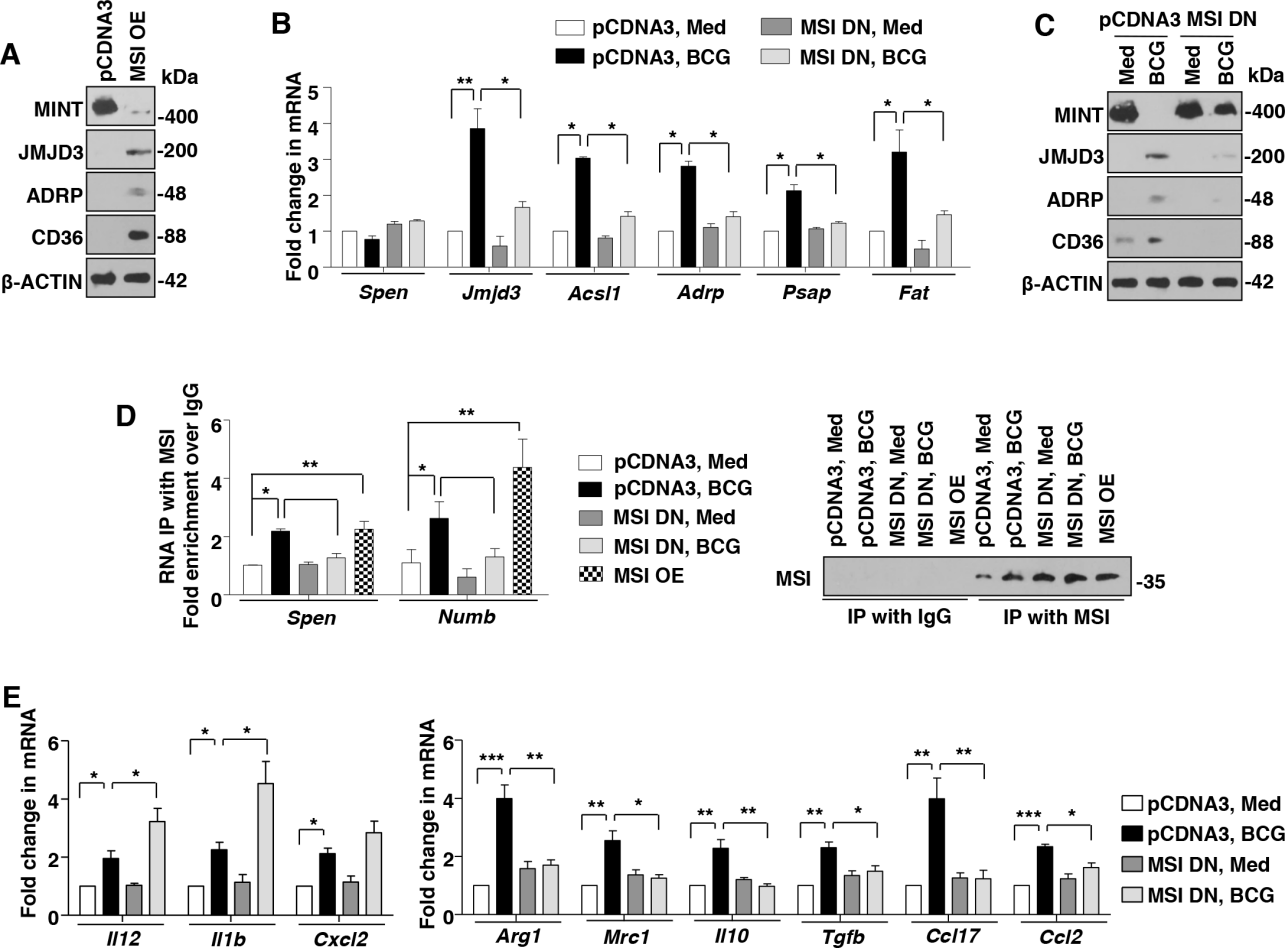
MSI regulates JMJD3 expression and concomitant immune responses by targeting MINT. (A-C) MSI1 OE- (A) or MSI1 DN-transfected (B and C) RAW 264.7 macrophages were analyzed for indicated genes in presence of BCG infection for 12 h by immunoblotting (A and C) or quantitative real-time RT-PCR (B). (D) MSI immunoprecipitates from RAW 264.7 cells transfected with MSI1 OE or MSI1 DN with BCG infection were analyzed for enrichment of MSI-binding region in the 3’UTR of *Spen* and *Numb* using quantitative real-time RT-PCR (left panel). Immunoprecipitated MSI was validated using immunoblotting (right panel). (E) Quantitative real-time RT-PCR for the indicated M1 and M2 markers from macrophages transiently transfected with MSI DN followed by infection with BCG for 12 h. All data represents the mean ± SEM for at least 3 independent experiments, *P < 0.05, **P < 0.005, ***P < 0.0005 (one-way ANOVA followed by Tukey’s multiple-comparisons test) and all blots are representative of 3 independent experiments. Med, medium; OE, overexpression; DN, dominant negative.

H37Rv, MDR-JAL2287, H37Ra and BCG, but not *M*. *smegmatis* infection of primary macrophages was found to exhibit elevated expression of MSI1 and MSI2 at both RNA and protein levels in a TLR2-dependent manner (Fig 5B–5F). Substantiating this observation, significant expression of MSI was found in the infected lungs as well as granuloma sections (Fig 5G and 5H). Further, BODIPY and ORO staining of RAW 264.7 macrophages expressing MSI overexpression construct (Fig 5I and S4A and S4B Fig) or MSI dominant negative (S4C and S4D Fig) and BODIPY analysis of primary macrophages transfected *Msi*-specific siRNA (Fig 5J and S4E Fig) suggested that MSI expression was crucial to mediate mycobacteria-induced FM generation. Supporting this observation, the genes regulating FM formation, *Jmjd3*, *Acsl1*, *Adrp*, *Psap* and *Fat* were positively regulated by MSI (Fig 6A, 6B and 6C). Interestingly, expression of MINT, a putative target of MSI, was not only suppressed in RAW 264.7 macrophages overexpressing MSI, but also rescued in macrophages expressing MSI dominant negative despite the infection (Fig 6A and 6C). To further validate the direct interaction of MINT with MSI, RNA IP experiments were performed. *Numb* is known target of MSI and was used as a positive control in the experiment [31]. Importantly, the MSI immunoprecipitates from RAW 264.7 macrophages infected with BCG or from RAW 264.7 macrophages overexpressing MSI showed significant enrichment of MSI binding region from 3’UTR of *Spen* and *Numb* (Fig 6D). However, BCG-induced enrichment of MSI binding region from *Spen* and *Numb* 3’UTR was severely abolished in macrophages expressing MSI dominant negative (Fig 6D). Together, these results establish that MINT is bonafide target of MSI. We further assessed the contribution of MSI in regulating the immune responses displayed by FMs. In accordance with JMJD3, MSI negatively regulated M1 markers like *Il12*, *Il1b* and *Cxcl2* (Fig 6E, left panel) and was necessary for mycobacterial infection-induced expression of M2 markers like *Arg1*, *Mrc1*, *Il10*, *Tgfb*, *Ccl17* and *Ccl2* (Fig 6E, right panel).

### NOTCH1-dependent expression of MSI and FM generation during mycobacterial infection

**Fig 7:**
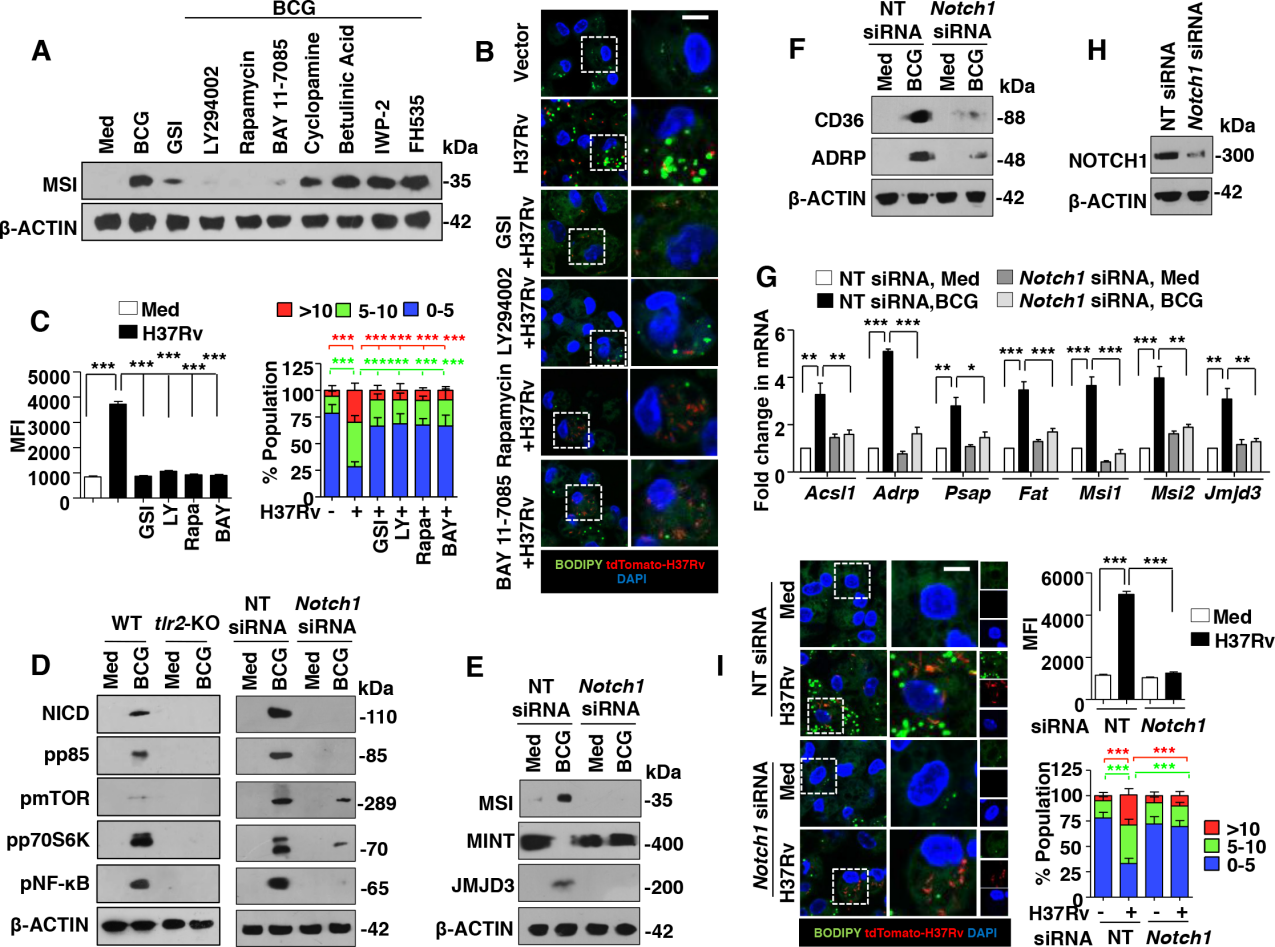
NOTCH1 signaling is essential for mycobacteria-mediated MSI expression and FM formation. (A) Mouse peritoneal macrophages were treated with specific inhibitors of NOTCH1 activation (GSI), PI3K (LY294002), mTOR (Rapamycin), NF-κB (BAY 11–7085), SHH signaling (Cyclopamine, Betulinic Acid) and WNT signaling (IWP-2, FH535) 60 min prior to 6 h infection with BCG. Immunoblotting for MSI was performed. (B and C) Peritoneal macrophages treated with specific inhibitors were infected with H37Rv expressing the red fluorescent protein tdTomato for 48 h. Representative IF images of macrophages with BODIPY-stained lipid droplets (B). Based on the IF images, MFIs were calculated (n = 100, each treatment) and plotted (C, left panel). Frequency of FMs was calculated by counting the population of cells expressing 0–5, 5–10 or >10 lipid bodies (n = 250–300) and plotted as a bar graph (C, right panel). (D) Activation analysis of NOTCH-PI3K-mTOR-NF-κB pathway by immunoblotting in peritoneal macrophages from WT or *tlr2*-KO mice (left panel) and RAW 264.7 cells transfected with *Notch1*-specific siRNAs (right panel) after 1 h of BCG infection. (E-H) Murine RAW 264.7 macrophages transfected with *Notch1*-specific siRNAs were infected with BCG for 12 h and analyzed for the expression of the indicated genes by immunoblotting (E and F) and quantitative real-time RT-PCR (G). Immunoblotting for NOTCH1 to validate *Notch1* siRNA (H). (I) IF imaging of BODIPY-stained lipid droplets in peritoneal macrophages transfected with NT or *Notch1* siRNA and infected with H37Rv expressing the red fluorescent protein tdTomato for 48 h (I, left panel). MFIs (top right panel) and frequency of FMs (bottom right panels) were calculated as mentioned in panel C. All data represents the mean ± SEM for at least 3 independent experiments, *P < 0.05, **P < 0.005, ***P < 0.0005 (one-way ANOVA followed by Tukey’s multiple-comparisons test) and all blots are representative of 3 independent experiments. Med, medium; WT, wild-type; KO, knockout; NT, non-targeting; MFI, mean fluorescence intensity. Bar, 5 μm; Original magnifications 63X.

**Fig 8:**
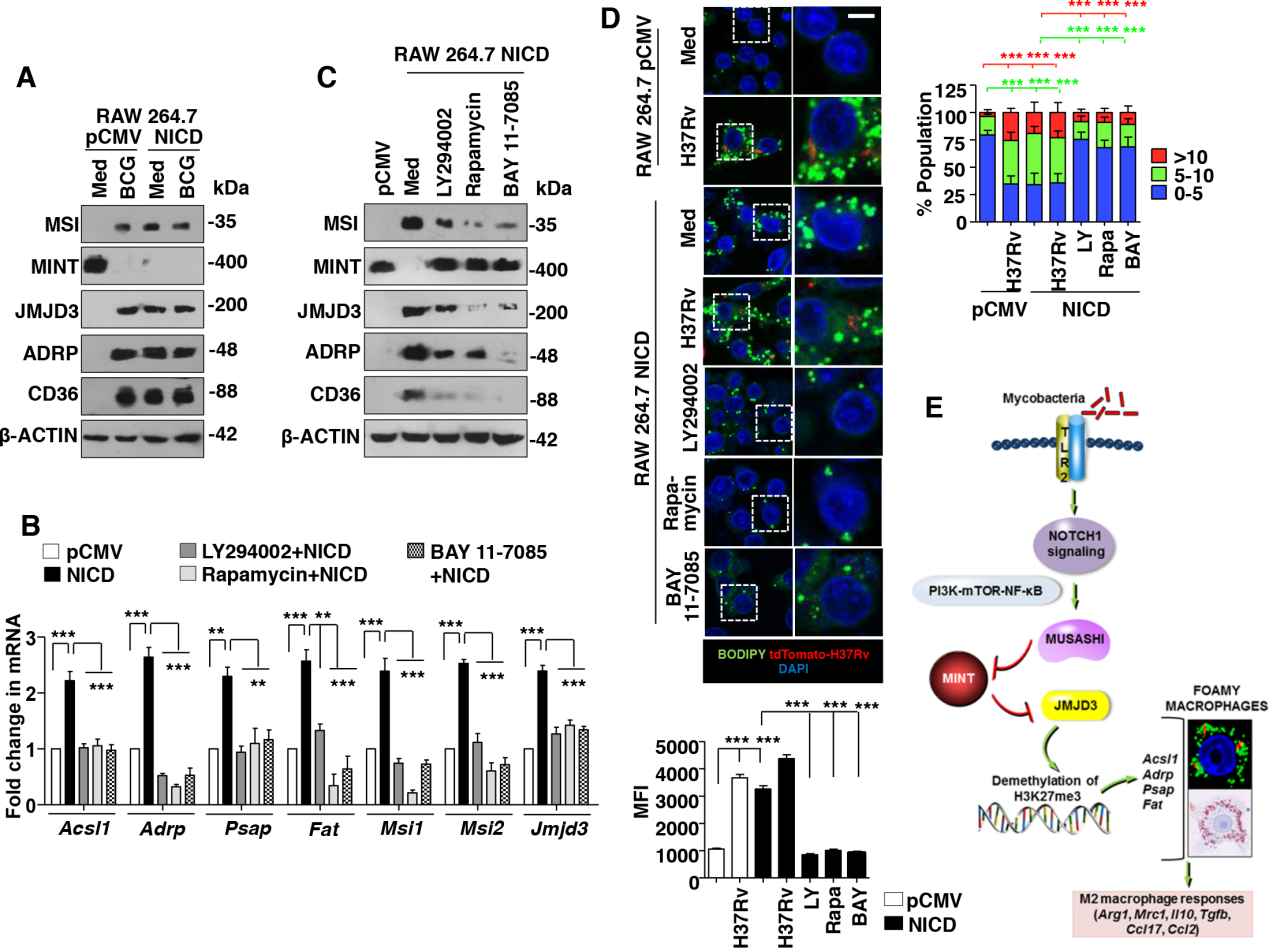
NOTCH1 regulates the FM formation via the PI3K-mTOR-NF-κB pathway. (A-C) Murine RAW 264.7 macrophages stably transfected with pCMV NICD (NICD) or pCMV alone (pCMV) were analyzed for the expression of the indicated genes either 12 h post BCG infection (A) or after treatment with the specific inhibitors of PI3K (LY294002), mTOR (Rapamycin), NF-κB (BAY 11–7085) (B and C) for 12 h. Immunoblotting (A, C) or quantitative real-time RT-PCR (B) analysis was performed. (D) RAW 264.7 macrophages stably expressing NICD were infected with H37Rv expressing the red fluorescent protein tdTomato for 48 h or treated with specific inhibitors. Representative IF images of macrophages with BODIPY-stained lipid droplets (top left panel). Based on the IF images, MFIs were calculated (n = 100, each treatment) and plotted (bottom left panel). Frequency of FMs was calculated by counting the population of cells expressing 0–5, 5–10 or >10 lipid bodies (n = 250–300) and plotted as a bar graph (top right panel). (E) Model presented in the study. All data represents the mean ± SEM for at least 3 independent experiments, **P < 0.005, ***P < 0.0005 (one-way ANOVA followed by Tukey’s multiple-comparisons test) and all blots are representative of 3 independent experiments. Med, medium; WT, wild-type; KO, knockout; NT, non-targeting; MFI, mean fluorescence intensity. Bar, 5 μm; Original magnifications 63X.

To establish the signaling link between TLR2 and MSI, a screen for various signaling pathways, previously known to be activated during mycobacterial infection [12–14, 33] was performed. Macrophages treated with specific inhibitors of NOTCH1 activation (GSI), PI3K (LY294002), mTOR (Rapamycin), NF-κB (BAY 11–7085), SHH signaling (Cyclopamine, Betulinic Acid) and WNT signaling (IWP-2, FH535) suggested a role for well-established NOTCH1-PI3K-mTOR-NF-κB pathway in regulating BCG-induced MSI expression (Fig 7A). Following inhibition of NOTCH1-PI3K-mTOR-NF-κB pathway with specific pharmacological inhibitors, the MFI and number of BODIPY-stained lipid bodies in H37Rv-infected primary macrophages were significantly reduced (Fig 7B and 7C). It has been well characterized that activation of NOTCH1 signaling is marked by cleavage of the intracellular domain of the NOTCH1 receptor to form NICD that transduces the downstream signaling. BCG infection-induced NOTCH1 signals via PI3K-mTOR-NF-κB cascade in macrophages in a TLR2-dependent manner (Fig 7D, left panel and [12, 33]). Further, BCG-infected macrophages expressing *Notch1*-specific siRNAs failed to activate the downstream PI3K-mTOR-NF-κB pathway (Fig 7D, right panel). In line with Fig 7A, BCG-regulated expression of MSI, MINT, JMJD3, genes associated with FM generation (Fig 7E–7H) and FM phenotype as assessed by BODIPY staining (Fig 7I) was found to be significantly reduced in macrophages expressing *Notch1*-specific siRNAs. These results underscore the NOTCH1 signaling functions that mediate mycobacteria-induced FM generation. Corroborating these results, RAW 264.7 macrophages stably expressing NICD exhibited the activation of PI3K-mTOR-NF-κB pathway (S5A Fig), induced comparable level of MSI, JMJD3, genes associated with FM generation and inhibited MINT expression (Fig 8A and S5B Fig). Additionally, similar to BCG-infected macrophages, NICD-expressing RAW 264.7 macrophages exhibited significant ORO staining (S5C Fig). Finally, inhibition of PI3K-mTOR-NF-κB pathway in RAW 264.7 macrophages stably expressing NICD significantly hampered their ability to induce the expression of MSI, JMJD3, genes associated with FM generation and FM phenotype (Fig 8B, 8C and 8D). Together, these results suggest the role for NOTCH1 signaling during LB generation and FM formation during mycobacterial infection.

## Discussion

In patients with *M*. *tuberculosis* infection, the bacilli were chiefly found to reside in the lipid-rich environment of FMs [34]. Further, both *in vitro* and *in vivo* studies in murine [28] and human granuloma models [34] have underscored the importance of the macrophage-derived FMs in regulating the course of mycobacterial pathogenesis. In accordance with previous observations [23, 34], in the current study, H37Rv, MDR-JAL2287, H37Ra and BCG, but not *M*. *smegmatis*, a saprophyte, were found to stimulate FM generation. *In vivo* generation of FMs in the granuloma was also observed. However, though several host [5, 23, 35] and bacterial [34, 35] components have been identified to regulate the infection-induced FM generation, no studies have attempted to unveil the epigenetic regulation that mediate LB and FM formation. We identified role for a histone demethylase, JMJD3, in orchestrating the mycobacterial infection-induced FM generation.

Functions of an inducible demethylase, JMJD3 has been implicated in case of several viral infections [36], bacterial effector functions [16, 37], inflammation [38] and M2 polarization [18]. Interestingly, pathogenic mycobacterial infection is well characterized for the generation of alternatively activated M2 macrophages, which could aid the bacterial survival and immune evasion [15, 20–22]. Importantly, many FM characteristic proteins like CD36, MSR1, lipoxygenases 5/15 etc are hallmark markers of M2 macrophages indicating a close link between M2 macrophages and FMs [23, 24]. In this context, the role for JMJD3 in mycobacteria-induced FMs was explored. JMJD3 was indeed, for the first time, found to coordinate the H37Rv-, MDR-JAL2287-, H37Ra- or BCG-induced FM formation and consequent M2 phenotype of the FMs. A recent report indicated an important role for JMJD3 during serum amyloid A-enhancement of oxidized LDL-induced macrophage FM generation [19]; however, there was no mechanism established in the study.

Of note, the genes that facilitate FM generation like *Acsl1*, *Adrp*, *Psap* and *Fat* exhibited elevated expression on infection with mycobacteria. Though the expression of *Msr1* and *Marco* was induced with infection, no significant changes were observed in *tlr2*-null macrophages or in the absence of *Jmjd3*, H3K27me3 demethylase. Hence, these genes were not pursued further. Interestingly, both virulent mycobacterial strains like H37Rv, MDR-JAL2287 and avirulent strains like H37Ra, BCG induced the robust expression of FM genes, but not *M*. *smegmatis*. We also found a robust expression of JMJD3-responsive FM genes on H37Rv or MDR-JAL2287 infection when compared to that with H37Ra and BCG. This could be attributed to the virulence characteristics of H37Rv and MDR-JAL2287. Further investigation on these aspects is underway. Also, the efflux coordinators, *Abca1* and *Abcg1* were downregulated or remained unchanged with H37Ra and BCG infection but were significantly induced during *M*. *smegmatis* infection. Though it needs further examination, increased ABCA1 and ABCG1 transporters in response to *M*. *smegmatis* could facilitate the efficient efflux of cholesterol from the infected macrophages and hence contribute to the reduced frequency of FMs during *M*. *smegmatis* infection. Together, in case of H37Rv, MDR-JAL2287, H37Ra and BCG, results indicate a concerted modulation of the lipid biosynthesis and uptake genes by a master regulator, JMJD3.

Further, mycobacteria-induced post-transcriptional regulator, MSI was found to target a negative regulator of JMJD3, MINT to facilitate the FM generation. In accordance to this observation, MINT was found to inhibit adipogenic differenciation [39] and inhibition of MINT could induce adipogenesis [40]. Likewise, we found suppression of MINT during pathogenic mycobacterial infection is requisite for JMJD3 expression and LB generation. Apart from a study that suggests MSI expression on infection with *Helicobacter pylori* that induces stemness and gastric cancer [41], no available reports have implicated the role for MSI during infection or inflammation. The functions of MSI are usually attributed to regulate cancer and development [32, 42]. Hence, a novel role for MSI during mycobacteria-responsive FM formation was elucidated in the current study. Importantly, while few M1 markers like *Il12*, *Il1b* and *Cxcl5* were negatively regulated by MSI-JMJD3 axis, characteristic M2 markers were directed by this pathway (in line with [18, 43]). The MSI-JMJD3-dependent M2 genes IL-10 and TGF-β including PGE_2_ which constitute FMs can regulate T cell responses including the generation and expansion of regulatory T cells [44, 45]. Thus, the identified pathway could largely contribute to the evasive responses during mycobacterial infection and suppression of such pathways during infection could confer stronger immunity.

Infection-induced NOTCH1-PI3K-mTOR-NF-κB signaling was found to mediate MSI expression. While mycobacterial infection was previously shown to induce TLR2-NOTCH1-PI3K-mTOR-NF-κB pathway to regulate immune responses [12, 33], different cancer studies have implicated NOTCH3-dependent MSI1 expression [46] and MSI1-dependent NOTCH activation [47]. Further, early activation of NOTCH1 signaling on mycobacterial infection [12, 33] is in line with the current observation of NOTCH1 signaling inducing MSI at early time points of infection. Together, TLR2-NOTCH1-dependent MSI induction was found to regulate the expression of a demethylase, JMJD3 by suppressing MINT. JMJD3 orchestrated the expression of the genes, *Acsl1*, *Adrp*, *Psap* and *Fat* to direct the formation of LBs and FMs (Fig 8E).

Confounding studies attribute both anti-bacterial and pro-bacterial functions of TLR2 during mycobacterial infection [15, 48–50]. Importantly, *tlr2*-null mice were previously reported to exhibit exaggerated immune responses to high dose (500 CFUs, as in the current study) mycobacterial infection but displayed dispersed granuloma, reduced bacterial clearance and succumbed to infection [51]. Elevated inflammatory phenotype was entailed to TLR2-dependent recruitment of Foxp3^+^ regulatory T cells to lungs that was compromised in *tlr2*-null mice [52]. In the current investigation, however, we found a pro-mycobacterial role for TLR2, as a requirement to establish the FMs in granuloma. Due to the fact that TLR2 effectuates multiple immune responses during mycobacterial infection, the exact contribution of TLR2-dependent JMJD3 *in vivo* in terms of regulating mycobacteria-induced immune responses needs to be assessed in *jmjd3*-null mice. Since loss of *jmjd3* causes perinatal lethality in mice [53], this study needs alternative strategies.

Our work has underscored the novel functions of epigenetic regulators like JMJD3 during mycobacteria-induced generation of a survival niche like FMs in granulomas and fine-tuning the concomitant immune responses. These regulators could be potential candidates for host-directed therapies against mycobacterial infection.

## Materials and Methods

### Cells, mice and bacteria

Primary macrophages were isolated from peritoneal exudates of C57BL/6J, C3H/HeJ and *tlr2*-KO mice that were purchased from The Jackson Laboratory and maintained in the Central Animal Facility, Indian Institute of Science (IISc). Briefly, mice were intraperitoneally injected with 1 ml of 8% Brewer thioglycollate. After 4 d of injection, mice were sacrificed and peritoneal cells were harvested by lavage from peritoneal cavity with ice-cold PBS. The cells were cultured in DMEM (Gibco-Invitrogen/Thermo Fisher Scientific) containing 10% FBS (Gibco-Invitrogen/Thermo Fisher Scientific) for 6 to 8 h and adherent cells were used as peritoneal macrophages. Murine RAW 264.7 macrophage-like cells obtained from the National Center for Cell Sciences, Pune, India. *M*. *tuberculosis* H37Rv and MDR-JAL2287 were kind research gifts from Dr. Kanury V.S. Rao, ICGEB, India. All studies involving virulent mycobacterial strains were carried out at the BSL-3 facility at Centre for Infectious Disease Research (CIDR), IISc. *M*. *bovis* BCG Pasteur 1173P2 was obtained from Pasteur Institute, Paris, France; *M*. *tuberculosis* H37Ra and *M*. *smegmatis* were kind research gifts from Dr. P. Ajitkumar, IISc, India. Bacteria were grown to mid-log phase and used at 10 multiplicity of infection (MOI) in all the experiments unless mentioned otherwise.

### Ethics statement

All studies involving mice and virulent mycobacterial strains were carried out after the approval from the Institutional Ethics Committee for animal experimentation as well as from Institutional Biosafety Committee. The animal care and use protocol adhered were approved by national guidelines of the Committee for the Purpose of Control and Supervision of Experiments on Animals (CPCSEA), Government of India.

### Reagents and antibodies

General laboratory chemicals were obtained from Sigma-Aldrich or Merck Millipore. Anti-β-ACTIN and anti-HA antibodies were purchased from Sigma-Aldrich. Anti-H3K27me3, anti-EZH2, anti-JMJD3, anti-MUSASHI (MSI), anti-NUMB, anti-NOTCH1, anti-Cleaved Notch1 (Val1744) (NICD), anti-Tyr485 p85/ Tyr199 p55 phospho-PI3K, anti-Ser2448 phospho-mTOR, anti-Thr389 phospho-p70S6K and anti-Ser536 phospho-NF-κB p65 were purchased from Cell Signaling Technology. Anti-ADRP, anti-CD36, anti-ABCA1, anti-SMRTe and anti-MINT (SPEN) were purchased from Santa Cruz Biotechnology, Inc. HRP conjugated anti-rabbit IgG and anti-mouse IgG and anti-rabbit DyLight 488 were obtained from Jackson ImmunoResearch. Fluorescein isothiocyanate (FITC)-conjugated monoclonal antibodies (mAbs) to mouse MHC class II, phycoerythrin (PE)-conjugated mAbs to mouse F4/80 were from BD Biosciences. Anti-mouse CD19-APC and CD3-FITC were from Imgenex. Ziehl-Neelsen (ZN) staining Kit was purchased from HiMedia and 4′,6-Diamidino-2-phenylindole dihydrochloride (DAPI) was from Sigma-Aldrich. BODIPY 493/503 (4,4-Difluoro-1,3,5,7,8-Pentamethyl-4-Bora-3a,4a-Diaza-*s*-Indacene) and HCS LipidTOX Red neutral lipid stain was from Molecular Probes (Invitrogen/Thermo Fisher Scientific).

### Treatment with pharmacological reagents

In all experiments, cells were treated with the given inhibitor (from Calbiochem) for 1 h before experimental treatments at following concentrations: GSI (10 μM), LY294002 (50 μM), Rapamycin (100 nM), BAY 11–7085 (10 μM), Cyclopamine (10 μM), Betulinic Acid (10 μM), IWP-2 (5 μM), FH535 β-CATENIN and TCF inhibtor (15 μM). DMSO at 0.1% concentration was used as the vehicle control. In all experiments involving pharmacological reagents, a tested concentration was used after careful titration experiments assessing the viability of the macrophages using the MTT (3-(4,5-Dimethylthiazol-2-yl)-2,5-diphenyltetrazolium bromide) assay.

### Stable transfection of RAW 264.7 cells

RAW 264.7 macrophages stably expressing NOTCH intracellular domain (NICD) were generated as described previously [12]. Briefly, RAW 264.7 cells were transfected with pCMV-NICD cDNA construct or pCMV alone using Lipofectamine 2000 (Invitrogen/Thermo Fisher Scientific). Cells were selected in G418 sulfate (400 μg/ml) and screened for NICD expression by immunoblotting as well as assessed for the expression of *Hes1* mRNA, a transcriptional target for NOTCH1 by quantitative real-time RT-PCR.

### Transient transfection studies

Transiently transfection of RAW 264.7 macrophages with 5 μg of dominant negative mutant forms of TLR2, MSI or overexpression constructs of JMJD3, MINT and MSI was performed using low m.w. polyethylenimine (Sigma-Aldrich). In case of experiments involving siRNA, RAW 264.7 macrophage cells were transfected with 100 nM siRNA. *Jmjd3*, *Acsl1*, *Adrp*, *Fat*, *Psap*, *Spen*, *Msi*, *Notch1*, non-targeting siRNA and siGLO Lamin A/C were obtained from Dharmacon as siGENOME SMARTpool reagents, which contain a pool of four different double-stranded RNA oligonucleotides. Transfection efficiency was found to be 70–80% in all the experiments as determined by counting the number of siGLO Lamin A/C positive cells in a microscopic field using fluorescent microscope. Further, 48 h post-transfection (for experiments with RAW 264.7 cells) or 24–36 h post-transfection (for experiments with peritoneal macrophages), the cells were treated or infected as indicated and processed for analysis.

### RNA isolation and quantitative real-time RT-PCR

Macrophages were treated or infected as indicated and total RNA from macrophages was isolated by TRI reagent (Sigma-Aldrich). 2 μg of total RNA was converted into cDNA using First strand cDNA synthesis kit (Applied Biological Materials Inc.). Quantitative real-time RT-PCR was performed using SYBR Green PCR mixture (KAPA Biosystems) for quantification of the target gene expression. All the experiments were repeated at least three times independently to ensure the reproducibility of the results. *Gapdh* was used as internal control. The primers used for quantitative real-time RT-PCR amplification are summarized in S1 Table.

### Immunoblotting

Immunoblotting was performed as previously mentioned elsewhere [15]. Infected or treated macrophages were lysed in RIPA buffer constituting 50 mM Tris-HCl (pH 7.4), 1% NP-40, 0.25% Sodium deoxycholate, 150 mM NaCl, 1 mM EDTA, 1 mM PMSF, 1 μg/ml of each aprotinin, leupeptin, pepstatin, 1 mM Na_3_VO_4_ and 1 mM NaF. Equal amount of protein from each cell lysate was resolved on a 12% SDS-polyacrylamide gel and transferred to polyvinylidene difluoride membranes (PVDF) (Millipore) by the semi-dry transfer (Bio-Rad) method. Nonspecific binding was blocked with 5% nonfat dry milk powder in TBST [20 mM Tris-HCl (pH 7.4), 137 mM NaCl, and 0.1% Tween 20] for 60 min. The blots were incubated overnight at 4°C with primary antibody followed by incubation with anti-rabbit-HRP or anti-mouse-HRP secondary antibody in 5% BSA for 2 h. After washing in TBST, the immunoblots were developed with enhanced chemiluminescence detection system (Perkin Elmer) as per manufacturer’s instructions. β-ACTIN was used as loading control.

### Chromatin Immunoprecipitation (ChIP) assay

ChIP assays were carried out using a protocol provided by Upstate Biotechnology and Sigma-Adrich with certain modifications. Briefly, macrophages were fixed with 3.6% formaldehyde for 15 min at room temperature followed by inactivation of formaldehyde with addition of 125 mM glycine. Nuclei were lysed in 0.1% SDS lysis buffer [50 mM Tris-HCl (pH 8.0), 200 mM NaCl, 10 mM HEPES (pH 6.5), 0.1% SDS, 10 mM EDTA, 0.5 mM EGTA, 1 mM PMSF, 1 μg/ml of each aprotinin, leupeptin, pepstatin, 1 mM Na_3_VO_4_ and 1 mM NaF]. Chromatin was sheared using Bioruptor Plus (Diagenode) at high power for 40 rounds of 30 sec pulse ON/45 sec OFF. Chromatin extracts containing DNA fragments with an average size of 500 bp were immunoprecipitated using JMJD3- or H3K27me3-specific antibodies or rabbit preimmune sera complexed with Protein A agarose beads (Bangalore Genei). Immunoprecipitated complexes were sequentially washed [Wash Buffer A: 50 mM Tris-HCl (pH 8.0), 500 mM NaCl, 1 mM EDTA, 1% Triton X-100, 0.1% Sodium deoxycholate, 0.1% SDS and protease/phosphatase inhibitors; Wash Buffer B: 50 mM Tris-HCl (pH 8.0), 1 mM EDTA, 250 mM LiCl, 0.5% NP-40, 0.5% Sodium deoxycholate and protease/phosphatase inhibitors; TE: 10 mM Tris-HCl (pH 8.0), 1 mM EDTA] and eluted in elution buffer [1% SDS, 0.1 M NaHCO_3_]. After treating the eluted samples with RNase A and Proteinase K, DNA was precipitated using phenol-chloroform-ethanol method. Purified DNA was analyzed by quantitative real time RT-PCR. All values in the test samples were normalized to amplification of the specific gene in Input and IgG pull down and represented as fold change in modification or enrichment. All ChIP experiments were repeated at least three times and the primers utilized are listed in S1 Table.

### H37Rv aerosol infection of mice

H37Rv was grown in Middlesbrook 7H9 medium (Difco) containing 0.2% glycerol, 0.05% Tween 80 and 10% ADC. Cultures were grown at 37°C to log phase. Culture was washed with PBS and passed 10 times each through 26-, 29- and 31-gauge needles to make single cell suspensions of H37Rv. C57BL/6 and *tlr2* null mice (n = 6, each group) were infected with 500 CFUs of H37Rv via aerosol using an aerosol chamber (Wisconsin-Madison). The pulmonary infection dose was confirmed by plating the homogenized lung tissue on Middlebrook 7H10 (Difco) agar plates. Post 8 weeks of aerosol infection, mice were sacrificed; lungs were collected, fixed overnight with 3.6% formaldehyde (Sigma-Aldrich) and processed for cryotomy or microtomy. Sections of fixed mice lungs were stained with hematoxylin and eosin to assess the pathology. Granulomas features were characterized and assigned different scores: with necrosis (Score = 5), without necrosis (Score = 2.5), with fibrosis (Score = 1). Total granuloma scores were calculated by multiplying the characterized feature score with the number of granuloma in each lung.

### *In vivo* murine BCG-induced granuloma model

C57BL/6 and *tlr2* null mice (n = 7, each group) were used for generating granulomas as previously described [3, 5, 28] with certain modifications. BCG (10^7^) were resuspended in 300 ml of ice-cold growth-factor reduced matrigel (Sigma-Aldrich). The mixture was injected sub-dermally into the skin fold at the scruff of the neck. Both sets of mice received either matrigel alone or BCG mixed matrigel. Granulomas were excised at 7 day post-inoculation and processed for cryotomy or microtomy.

### Cryosection preparation

The excised granuloma was rapidly frozen in liquid nitrogen in the optimal cutting temperature (OCT) media (Jung, Leica). Cryosections of 10–15 μm were taken in Leica CM 1510 S or Leica CM 3050 S cryostat (both Leica) with the tissue embedded in OCT onto the glass slides and stored at -80°C.

### Oil Red O (ORO) staining

Cells/cryosection tissues were fixed in 3.6% formaldehyde for 15 min. After PBS wash, the cells/tissues were rinsed in 60% isopropanol for 10 min and stained with 0.5% ORO for 15 min. After cleaning the cells/tissues with 60% isopropanol, they were either counterstained with haematoxylin and viewed under 100X or 20X in light microscope or washed in 100% isopropanol for colorimetric analysis at 510 nm.

### Lipid body staining and analysis

Lipid body staining was performed using a protocol provided by the manufacturer with certain modifications. The cells/cryosection tissues were fixed with 3.6% formaldehyde for 45 min. After 3 washes with PBS, the cells/cryosection tissues were stained with 10 μg/ml BODIPY 493/503 or 1X HCS LipidTOX Red neutral lipid stain for 30 min in dark. After 3 washes with PBS, the cells/sections were stained with DAPI and mounted on a slide with glycerol as the medium. Confocal images (Z-stacks) were taken on Zeiss LSM 710 Meta confocal laser scanning microscope (Carl Zeiss AG) using a plan-Apochromat 63X/1.4 Oil DIC objective (Carl Zeiss AG) and images were analyzed using ZEN 2009 and ImageJ softwares. The lipid bodies were counted in over 250 cells from different fields. Frequency of populations with different number of lipid bodies (0–5 in blue, 5–10 in green, >10 in red) were plotted in terms of percentage. Statistical significance is represented in green and red. Green line represents the significance analysis of populations with “5–10” lipid bodies between indicated treatments and red represents the significance analysis of populations with “>10” lipid bodies. For the MFI analysis, ImageJ was utilized to calculate the maximum intensity projections of the Z-stacks. Using free hand selection tool, cells were selected to measure the area-integrated intensity and mean grey value. The area around the cells without fluorescence was used to calculate the background values. Corrected Total Cell Fluorescence (CTCF) was calculated using the following formula: CTCF = Integrated intensity–(area of selected cell X Mean florescence of background reading).

### BODIPY 493/503 analysis of the lungs

Uninfected and H37Rv-infected lungs were finely chopped and digested in 5 ml RPMI with 5% FBS containing 150 U/ml Collagenase IV (HiMedia) and 50 U/ml DNaseI (Thermo Fisher Scientific) at 37°C in shaking condition for 1 h. The cell suspension was passed through 40 μm cell strainer and pelleted at 1500 rpm for 10 min. The cells were resuspended in RBC lysis buffer. Post lysis, the cells were washed with PBS. Obtained cell suspension was fixed using 3.6% formaldehyde solution for 30 min. After 3 washes with PBS, cells were resuspended in PBS containing 2 μg/ml BODIPY 493/503 and incubated for 30 min at room temperature in dark under mild rocking condition. After thorough washes with PBS, cells were analysed by flow cytometry wherein 1 lakh events were recorded for each sample acquired in BD FACSCanto II. The data was analyzed using FACSDiva software (BD Biosciences) and WinMDI Version 2.8.

### Immunofluorescence (IF)

In case of MINT validation experiments, RAW 264.7 macrophages-transfected with MINT-EGFP were seeded on to coverslips and incubated. The cells were fixed with 3.6% formaldehyde for 15 min at room temperature and the coverslips were mounted on a slide with glycerol. For IF of the cryosections, frozen sections were thawed to room temperature and fixed with 3.6% formaldehyde. After blocking with 2% BSA containing saponin, the sections were stained for specific antibodies at 4°C overnight. The sections were incubated with DyLight 488-conjugated secondary antibody for 2 h and nuclei stained with DAPI. A coverslip was mounted on the section with glycerol as the medium. Confocal images were taken on Zeiss LSM 710 Meta confocal laser scanning microscope (Carl Zeiss AG) using a plan-Apochromat 63X/1.4 Oil DIC objective (Carl Zeiss AG) and images were analyzed using ZEN 2009 software.

### Immunohistochemistry (IHC)

Microtome sections (4 μm) were obtained from 3.6% formaldehyde-fixed, decalcified, and paraffin-embedded tissues using Leica RM2245 microtome (Leica). These sections were first deparaffinized, subjected to antigen retrieval by boiling in 10 mM citrate buffer (pH 6.0) for 10 min, treated with 1% H_2_O_2_ for 10 min, and blocked with 5% BSA for 1 h at room temperature. The tissue sections were further incubated with primary antibodies overnight. After incubation with anti-rabbit HRP-conjugated secondary antibody for 90 min, sections were stained with 0.05% diaminobenzidine (Sigma-Aldrich) in 0.03% H_2_O_2_ solution and counterstained with hematoxylin, dehydrated and mounted. Stained tissue sections were imaged with Axio Scope.A1 microscope (Zeiss) at indicated magnification. All experiments were performed with appropriate isotype-matched control antibodies.

### Ziehl-Neelsen (ZN) staining

Cryosections were fixed, paraffin-embedded sections were deparaffinized and hydrated to distilled water. The sections were stained with hot Carbol fuchsin solution for 5 min. The sectioned were washed in running water and destained with 1% acid alcohol. After washing with running water, the sections were counter-stained with methylene blue for 30 sec. The sections were washed and dried. The images were acquired with Axio Scope.A1 microscope (Zeiss) at indicated magnification.

### RNA Immunoprecipitation (RNA IP)

Macrophages were lysed in 300 μl of complete polysomal lysis buffer [5 mM MgCl_2_, 0.1 M KCl, 0.5% NP40, 0.01M HEPES pH 7.5]. Total cell lysate (200 μg) was diluted to 500 μl using complete polysomal lysis buffer for IP. 50 μl of anti-mouse IgG precleared lysate was used as Input for the experiment and total RNA was isolated as described earlier. Rest of the precleared lysate was incubated with 1 μg anti-mouse IgG or anti-MSI prebound Protein A beads overnight. Further, beads were washed in complete polysomal lysis buffer and 25% of the beads were eluted in 5X Laemmli buffer for immunoblotting with anti-MSI. Remaining beads were eluted in TRI reagent and processed for RNA isolation as described earlier. 500 ng of the RNA was converted into cDNA and quantitative real time RT-PCR was performed to analyze *Spen* and *Numb*. The primers used are listed in S1 Table. *Gapdh* from Input was utilized for normalization.

### Statistical analysis

Levels of significance for comparison between samples were determined by the Student’s *t*-test distribution and one-way ANOVA followed by Tukey’s multiple-comparisons. The data in the graphs are expressed as the mean ± S.E for the values from at least 3 or more independent experiments and *P* values < 0.05 were defined as significant. GraphPad Prism 5.0 software (GraphPad Software) was used for all the statistical analysis.

## Supporting Information

S1 Fig TLR2- and JMJD3-dependent FM generation during mycobacterial infection. (A) Murine RAW 264.7 macrophages were transiently transfected with TLR2 DN and infected with the indicated bacteria (H37Ra: *M*. *tuberculosis* H37Ra; BCG: *M*. *bovis* BCG; MS: *M*. *smegmatis*) for 48 h. Representative images of cells stained with Oil Red O (ORO) (left panel) and extracted ORO was measured at OD_510_ (right panel). (B) IF imaging of BODIPY-stained lipid droplets in WT or *tlr2*-null peritoneal macrophages infected with the indicated bacteria for 48 h (left top panel). Based on the IF images, MFIs were calculated (n = 100, each treatment) and plotted (left bottom panel). Frequency of FMs was calculated by counting the population of cells expressing 0–5, 5–10 or >10 lipid bodies (n = 250–300) and plotted as a bar graph (right panel). (C) Murine RAW 264.7 macrophages were infected with 5 MOI of H37Rv or MDR-JAL2287 for 48 h. Representative images of cells stained with ORO (top panel) and extracted ORO was measured at OD_510_ (bottom panel). (D-G) RAW 264.7 cells transiently transfected with JMJD3-HA (D and E) or NT or *Jmjd3* siRNA (F and G) were infected with BCG for 48 h. ORO staining (D and F, left panels) and the extracted ORO at OD_510_ (D and F, right panels) was performed. Confirmatory blot for JMJD3-HA construct (E) and *Jmjd3*-specific siRNA (G). All data represents the mean ± SEM for at least 3 independent experiments, ns = not significant, *P < 0.05, **P < 0.005, ***P < 0.0005 (one-way ANOVA followed by Tukey’s multiple-comparisons test except for two-tailed paired Student’s *t*-test in D). Med, medium; DN, dominant negative; WT, wild-type; KO, knockout; NT, non-targeting; MFI, mean fluorescence intensity. Bar, 5 μm; Original magnifications 100X in A, C, D, F and 63X in B.
(TIF)

S2 Fig JMJD3 and genes associated with FM generation regulate M1/M2 genes. (A) RAW 264.7 macrophages transiently transfected NT or *Jmjd3* siRNA were infected with BCG for 12 h. Quantitative real-time RT-PCR for the indicated M1 markers. (B) Transcript levels analysis of the selected genes involved during FM formation in peritoneal macrophages infected with the indicated bacteria for 12 h. (C) Peritoneal macrophages from WT or *tlr2*-null mice were infected with BCG for 12 h. H3K27me3 modification (upper panel) and JMJD3 recruitment (lower panel) at promoters of M2 markers were evaluated by ChIP. (D) siRNA validation of the selected genes involved during FM formation by immunoblotting and quantitative real-time RT-PCR. (E) RAW 264.7 macrophages transiently transfected NT or *Acsl1+Adrp+Psap+Fat* siRNA were infected with BCG for 12 h. Quantitative real-time RT-PCR for the indicated M2 markers. All data represents the mean ± SEM for at least 3 independent experiments, ns = not significant, *P < 0.05, **P < 0.005, ***P < 0.0005 (one-way ANOVA followed by Tukey’s multiple-comparisons). Med, medium; NT, non-targeting; WT, wild-type; KO, knockout.
(TIF)

S3 Fig TLR2-dependent FM generation in mouse granulomas. (A) WT or *tlr2*-null mice were infected by aerosol inhalation of 500 CFUs of H37Rv (n = 6 in each group, two independent experiment). Pulmonary pathology was recorded after 8 weeks of infection; arrows indicate the presence of granuloma structures. (B) Cryosections of the lung tissues were stained with ORO. Representative images (left panel; Original magnifications 20X) and extracted ORO at OD_510_ (right panel). (C) BCG along with matrigel was injected to the scruff of the WT or *tlr2*-null mice to induce granuloma formation (n = 7 in each group). Representative immunofluorescence images stained for B cell (CD19) and T cell (CD3) markers or macrophage markers (F4/80, MHC-II) in the cryosections of the excised granuloma from BCG-infected WT mice. Formaldehyde-fixed, paraffin-embedded granuloma sections from BCG-infected WT mice were stained for acid-fast bacteria by Ziehl-Neelsen method (lower most panel). (D) Cryosections of the excised granuloma from WT and *tlr2*-KO mice were stained with ORO. Representative images (upper panel; Original magnifications 20X) and extracted ORO at OD_510_ (lower panel). (E) IF with cryosections of the lungs (left 4 panels)/ granuloma (right 4 panels) from WT and *tlr2*-KO mice was performed to assess the *in vivo* expression of JMJD3, ADRP and CD36. Representative images are shown here (n = 6). Original magnifications indicated on the images. ORO OD data represents the mean ± SEM, *P < 0.05, **P < 0.005, ***P < 0.0005 (one-way ANOVA followed by Tukey’s multiple-comparisons). WT, wild-type; KO, knockout. Original magnifications and scale are indicated on the images.
(TIF)

S4 Fig MSI regulates FM formation. (A-D) RAW 264.7 cells were transiently transfected with MSI1 OE (A and B) or MSI1 DN (C and D). BCG infection was for 48 h in panel C and 12 h in panel D. Representative images of cells stained with ORO (A and C, left panels) and the extracted ORO at OD_510_ (A and C, right panels). Immunoblotting of MSI and its target gene NUMB to validate the OE and DN constructs (B and D). (E) *Msi* siRNA-transfected RAW 264.7 macrophages were analyzed for MSI and its target gene NUMB in the presence of BCG infection for 12 h by immunoblotting. All data represents the mean ± SEM for at least 3 independent experiments, *P < 0.05 (two-tailed paired Student’s *t*-test in A and one-way ANOVA followed by Tukey’s multiple-comparisons test in C) and all blots are representative of 3 independent experiments. Med, medium; OE, overexpression; DN, dominant negative; NT, non-targeting. Bar, 5 μm; Original magnifications 100X.
(TIF)

S5 Fig NOTCH1 mediates FM generation. (A-C) Murine RAW 264.7 macrophages stably transfected with pCMV NICD (NICD) or pCMV alone (pCMV) were infected with BCG for 1 h (A), 12 h (B) or 48 h (C). Expression of the indicated genes was analyzed by immunoblotting (A) or quantitative real-time RT-PCR (B). ORO staining (C, left panel) and the extracted ORO at OD_510_ (C, right panel) was performed. All data represents the mean ± SEM for at least 3 independent experiments, *P < 0.05, **P < 0.005, ***P < 0.0005 (one-way ANOVA followed by Tukey’s multiple-comparisons) and all blots are representative of 3 independent experiments. Med, medium. Bar, 5 μm; Original magnifications 100X.
(TIF)

S1 Table Primers used in the study. (DOC)
