# Supplementary figures and images for: MUSASHI-Mediated Expression of JMJD3, a H3K27me3 Demethylase, Is Involved in Foamy Macrophage Generation during Mycobacterial Infection
Source: PLoS Pathog. 2016 Aug 17;12(8):e1005814. doi: 10.1371/journal.ppat.1005814 (PMC4988650; doi:10.1371/journal.ppat.1005814)

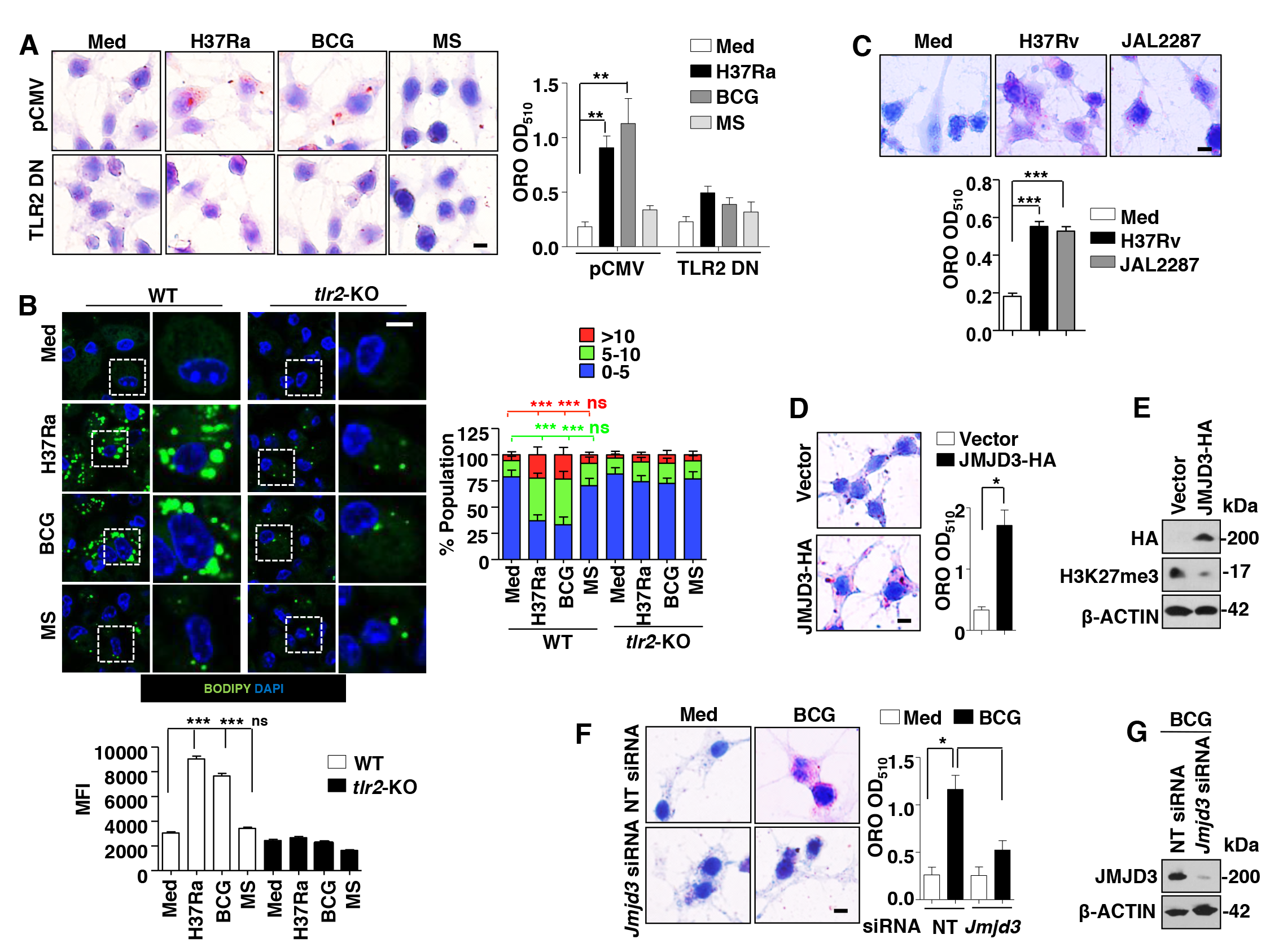

Supplement: S1 Fig — (A) Murine RAW 264.7 macrophages were transiently transfected with TLR2 DN and infected with the indicated bacteria (H37Ra: M. tuberculosis H37Ra; BCG: M. bovis BCG; MS: M. smegmatis) for 48 h. Representative images of cells stained with Oil Red O (ORO) (left panel) and extracted ORO was measured at OD510 (right panel). (B) IF imaging of BODIPY-stained lipid droplets in WT or tlr2-null peritoneal macrophages infected with the indicated bacteria for 48 h (left top panel). Based on the IF images, MFIs were calculated (n = 100, each treatment) and plotted (left bottom panel). Frequency of FMs was calculated by counting the population of cells expressing 0–5, 5–10 or >10 lipid bodies (n = 250–300) and plotted as a bar graph (right panel). (C) Murine RAW 264.7 macrophages were infected with 5 MOI of H37Rv or MDR-JAL2287 for 48 h. Representative images of cells stained with ORO (top panel) and extracted ORO was measured at OD510 (bottom panel). (D-G) RAW 264.7 cells transiently transfected with JMJD3-HA (D and E) or NT or Jmjd3 siRNA (F and G) were infected with BCG for 48 h. ORO staining (D and F, left panels) and the extracted ORO at OD510 (D and F, right panels) was performed. Confirmatory blot for JMJD3-HA construct (E) and Jmjd3-specific siRNA (G). All data represents the mean ± SEM for at least 3 independent experiments, ns = not significant, *P < 0.05, **P < 0.005, ***P < 0.0005 (one-way ANOVA followed by Tukey’s multiple-comparisons test except for two-tailed paired Student’s t-test in D). Med, medium; DN, dominant negative; WT, wild-type; KO, knockout; NT, non-targeting; MFI, mean fluorescence intensity. Bar, 5 μm; Original magnifications 100X in A, C, D, F and 63X in B. (TIF) [file ppat.1005814.s001.tif]

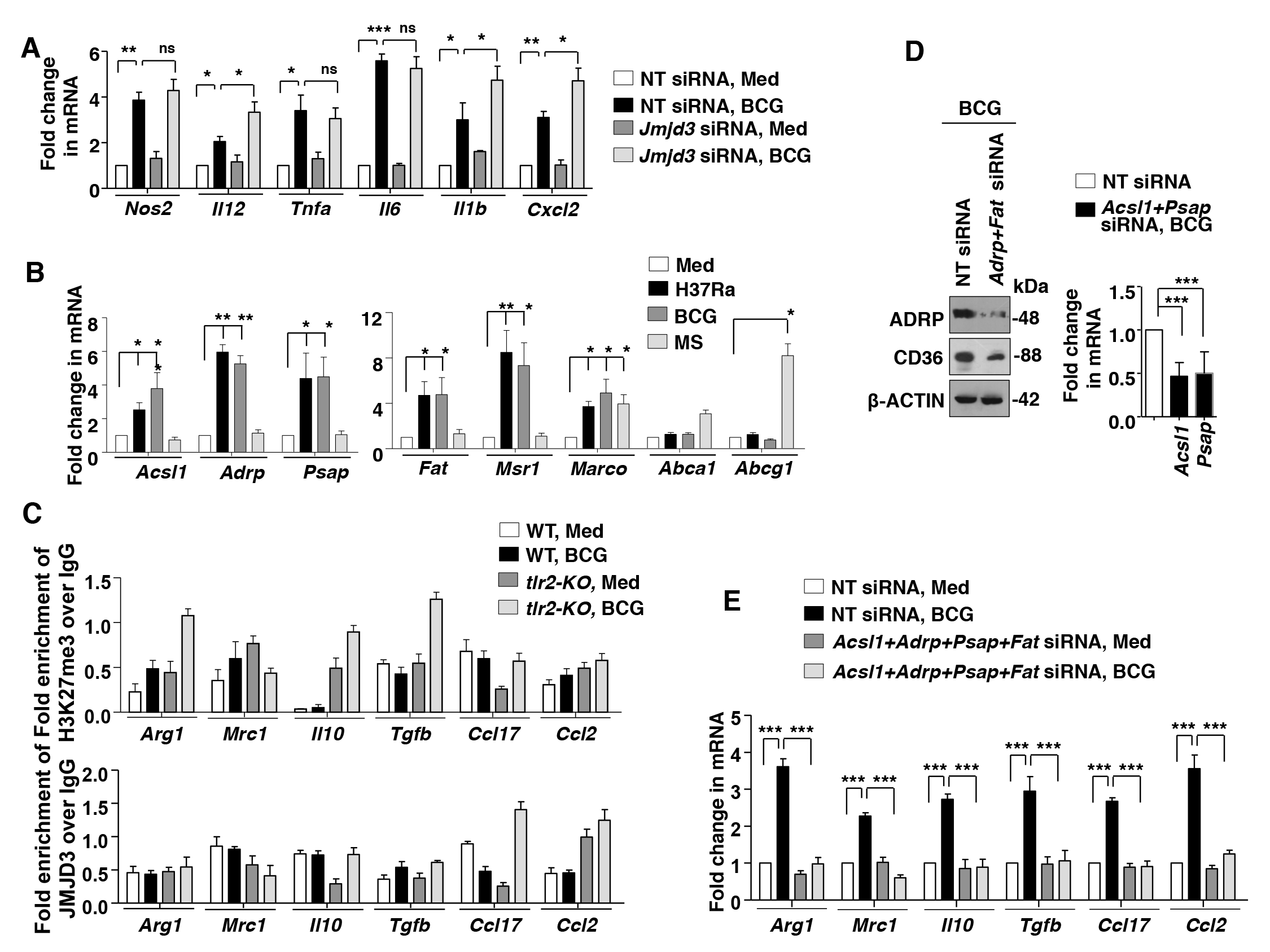

Supplement: S2 Fig — (A) RAW 264.7 macrophages transiently transfected NT or Jmjd3 siRNA were infected with BCG for 12 h. Quantitative real-time RT-PCR for the indicated M1 markers. (B) Transcript levels analysis of the selected genes involved during FM formation in peritoneal macrophages infected with the indicated bacteria for 12 h. (C) Peritoneal macrophages from WT or tlr2-null mice were infected with BCG for 12 h. H3K27me3 modification (upper panel) and JMJD3 recruitment (lower panel) at promoters of M2 markers were evaluated by ChIP. (D) siRNA validation of the selected genes involved during FM formation by immunoblotting and quantitative real-time RT-PCR. (E) RAW 264.7 macrophages transiently transfected NT or Acsl1+Adrp+Psap+Fat siRNA were infected with BCG for 12 h. Quantitative real-time RT-PCR for the indicated M2 markers. All data represents the mean ± SEM for at least 3 independent experiments, ns = not significant, *P < 0.05, **P < 0.005, ***P < 0.0005 (one-way ANOVA followed by Tukey’s multiple-comparisons). Med, medium; NT, non-targeting; WT, wild-type; KO, knockout. (TIF) [file ppat.1005814.s002.tif]

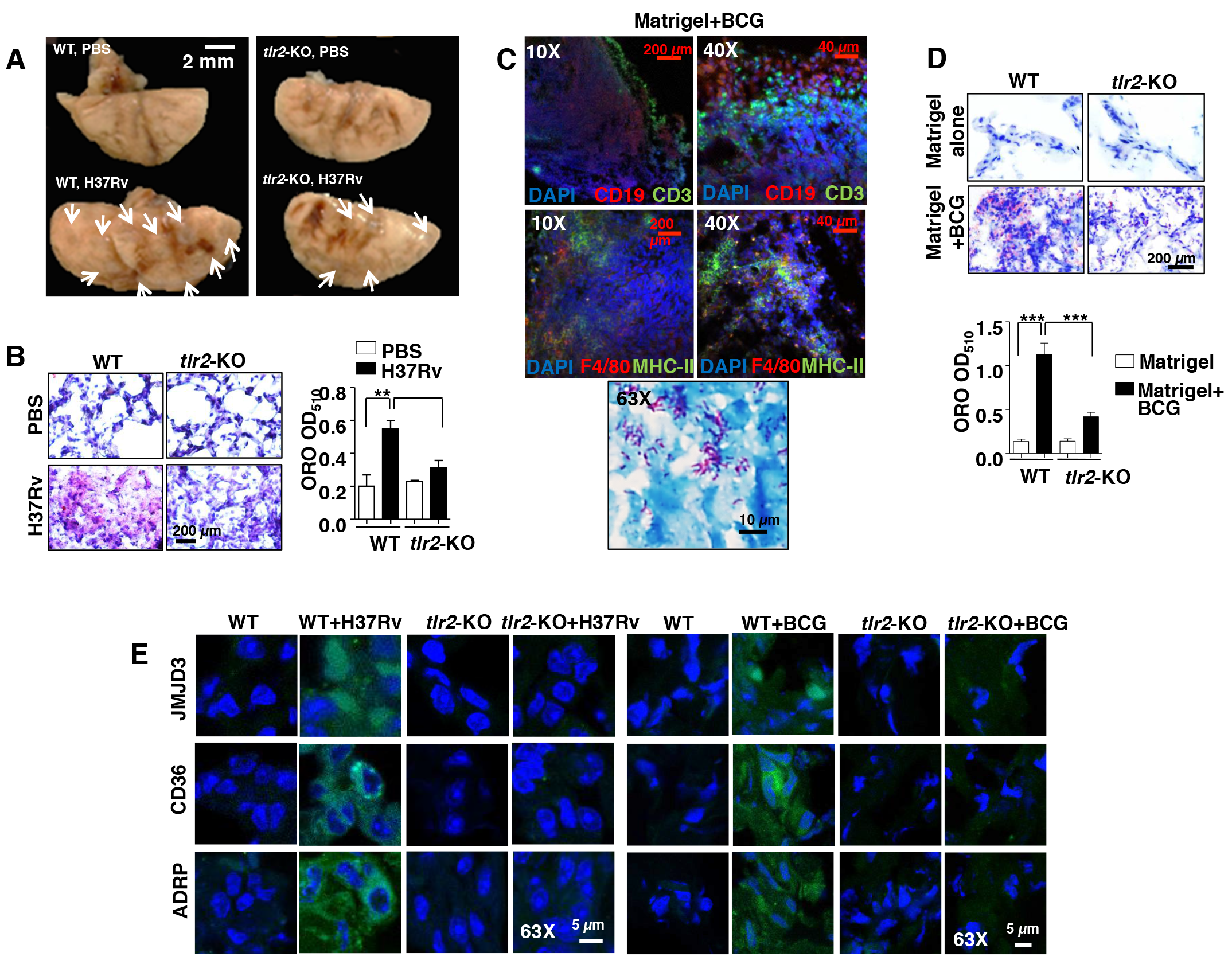

Supplement: S3 Fig — (A) WT or tlr2-null mice were infected by aerosol inhalation of 500 CFUs of H37Rv (n = 6 in each group, two independent experiment). Pulmonary pathology was recorded after 8 weeks of infection; arrows indicate the presence of granuloma structures. (B) Cryosections of the lung tissues were stained with ORO. Representative images (left panel; Original magnifications 20X) and extracted ORO at OD510 (right panel). (C) BCG along with matrigel was injected to the scruff of the WT or tlr2-null mice to induce granuloma formation (n = 7 in each group). Representative immunofluorescence images stained for B cell (CD19) and T cell (CD3) markers or macrophage markers (F4/80, MHC-II) in the cryosections of the excised granuloma from BCG-infected WT mice. Formaldehyde-fixed, paraffin-embedded granuloma sections from BCG-infected WT mice were stained for acid-fast bacteria by Ziehl-Neelsen method (lower most panel). (D) Cryosections of the excised granuloma from WT and tlr2-KO mice were stained with ORO. Representative images (upper panel; Original magnifications 20X) and extracted ORO at OD510 (lower panel). (E) IF with cryosections of the lungs (left 4 panels)/ granuloma (right 4 panels) from WT and tlr2-KO mice was performed to assess the in vivo expression of JMJD3, ADRP and CD36. Representative images are shown here (n = 6). Original magnifications indicated on the images. ORO OD data represents the mean ± SEM, *P < 0.05, **P < 0.005, ***P < 0.0005 (one-way ANOVA followed by Tukey’s multiple-comparisons). WT, wild-type; KO, knockout. Original magnifications and scale are indicated on the images. (TIF) [file ppat.1005814.s003.tif]

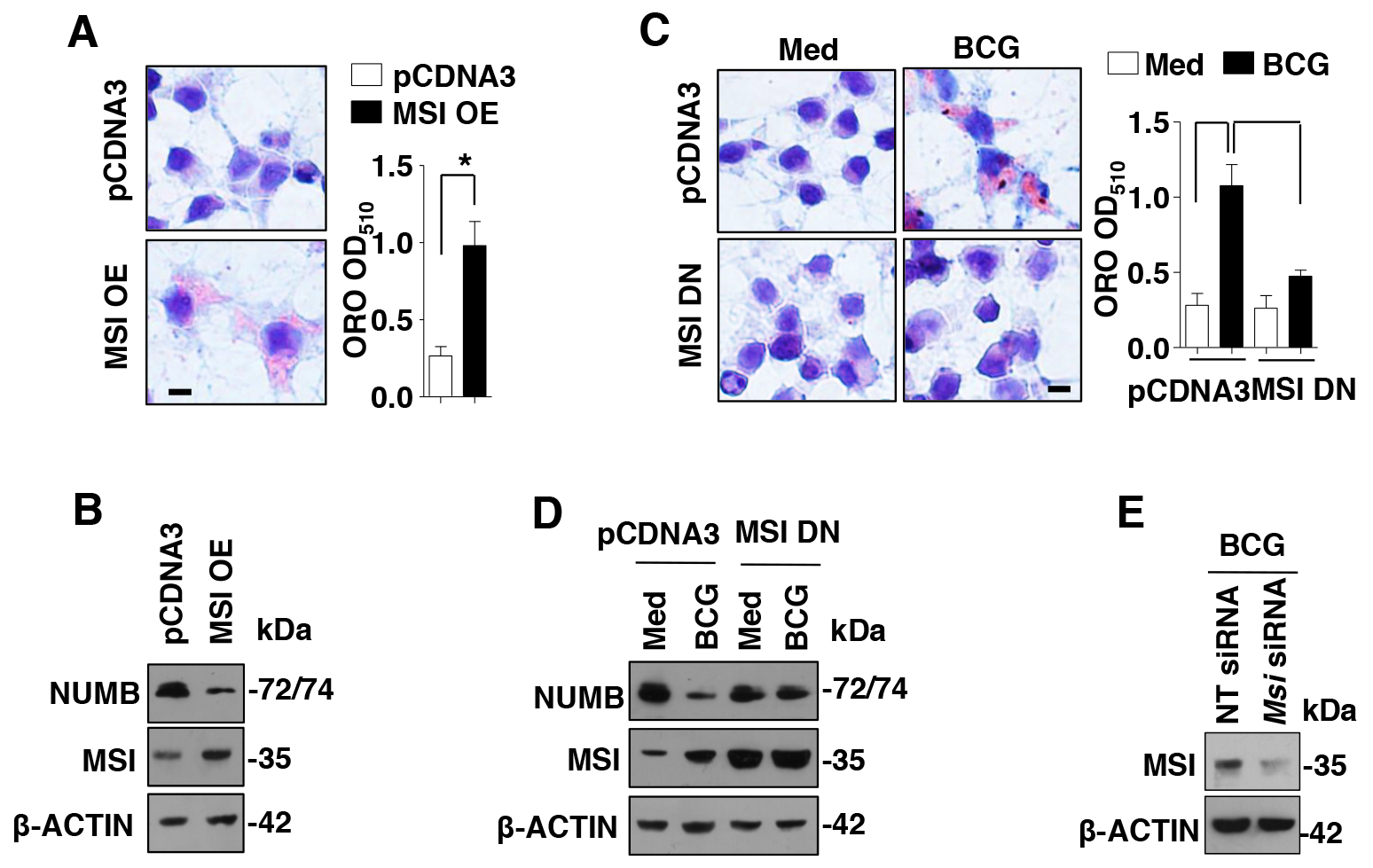

Supplement: S4 Fig — (A-D) RAW 264.7 cells were transiently transfected with MSI1 OE (A and B) or MSI1 DN (C and D). BCG infection was for 48 h in panel C and 12 h in panel D. Representative images of cells stained with ORO (A and C, left panels) and the extracted ORO at OD510 (A and C, right panels). Immunoblotting of MSI and its target gene NUMB to validate the OE and DN constructs (B and D). (E) Msi siRNA-transfected RAW 264.7 macrophages were analyzed for MSI and its target gene NUMB in the presence of BCG infection for 12 h by immunoblotting. All data represents the mean ± SEM for at least 3 independent experiments, *P < 0.05 (two-tailed paired Student’s t-test in A and one-way ANOVA followed by Tukey’s multiple-comparisons test in C) and all blots are representative of 3 independent experiments. Med, medium; OE, overexpression; DN, dominant negative; NT, non-targeting. Bar, 5 μm; Original magnifications 100X. (TIF) [file ppat.1005814.s004.tif]

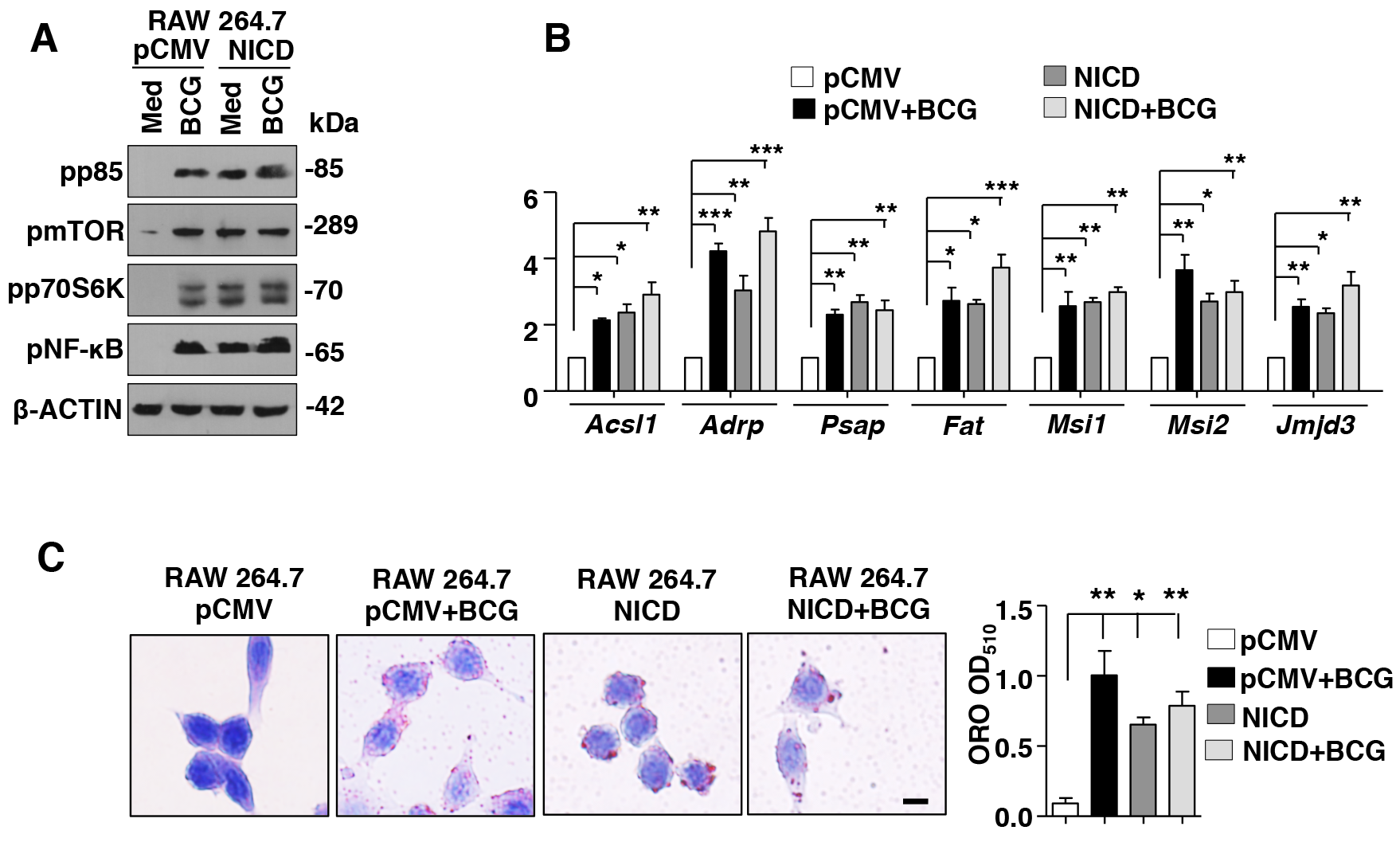

Supplement: S5 Fig — (A-C) Murine RAW 264.7 macrophages stably transfected with pCMV NICD (NICD) or pCMV alone (pCMV) were infected with BCG for 1 h (A), 12 h (B) or 48 h (C). Expression of the indicated genes was analyzed by immunoblotting (A) or quantitative real-time RT-PCR (B). ORO staining (C, left panel) and the extracted ORO at OD510 (C, right panel) was performed. All data represents the mean ± SEM for at least 3 independent experiments, *P < 0.05, **P < 0.005, ***P < 0.0005 (one-way ANOVA followed by Tukey’s multiple-comparisons) and all blots are representative of 3 independent experiments. Med, medium. Bar, 5 μm; Original magnifications 100X. (TIF) [file ppat.1005814.s005.tif]
