# Supplementary material for: MUSASHI-Mediated Expression of JMJD3, a H3K27me3 Demethylase, Is Involved in Foamy Macrophage Generation during Mycobacterial Infection
Source: PLoS Pathog. 2016 Aug 17;12(8):e1005814. doi: 10.1371/journal.ppat.1005814 (PMC4988650; doi:10.1371/journal.ppat.1005814)
Supplement: S1 Table — (DOC) [file ppat.1005814.s006.doc]

S1 Table. Primers used in the study.

| **For quantitative real-time RT-PCR** | | | |
| --- | --- | --- | --- |
| 1 | m *Gapdh* forward | 5’-gagccaaacgggtcatcatct-3’ | |
|  | m *Gapdh* reverse | 5’-gaggggccatccacagtctt-3’ | |
| 2 | m *Jmjd3* forward | 5’-tgaagaacgtcaagtccattgtg-3’ | |
|  | m *Jmjd3* reverse | 5’-tcccgctgtacctgacagt-3’ | |
| 3 | m *Acsl1* forward | 5’-gtcctgggcacagaagagag-3’ | |
|  | m *Acsl1* reverse | 5’-gtcagaaggccgttgtcaat-3’ | |
| 4 | m *Adrp* forward | 5’-ggagtggaagagaagcatcg-3’ | |
|  | m *Adrp* reverse | 5’-tggcatgtagtctggagctg-3’ | |
| 5 | m *Psap* forward | 5’-gatcctgccagaaccaagtg-3’ | |
|  | m *Psap* reverse | 5’-ctcaagtgcctccaccaact-3’ | |
| 6 | m *Fat* forward | 5’-cagtcggagacatgcttattgag-3’ | |
|  | m *Fat* reverse | 5’-tttgccacgtcatctgggttt-3’ | |
| 7 | m *Msr1* forward | 5’-ttcactggatgcaatctccaag-3’ | |
|  | m *Msr1* reverse | 5’-ctggacttctgctgatactttgt-3’ | |
| 8 | m *Marco* forward | 5’-acagagccgattttgaccaag-3’ | |
|  | m *Marco* reverse | 5’-cagcagtgcagtacctgcc-3’ | |
| 9 | m *Abca1* forward | 5’-aaaaccgcagacatccttcag-3’ | |
|  | m *Abca1* reverse | 5’-cataccgaaactcgttcaccc-3’ | |
| 10 | m *Abcg1* forward | 5’-gtggatgaggttgagacagacc-3’ | |
|  | m *Abcg1* reverse | 5’-cctcgggtacagagtaggaaag-3’ | |
| 11 | m *Mint* forward | 5’-gggctctattacacttctcgga-3’ | |
|  | m *Mint* reverse | 5’-tgtgttggtaactgcgctctg-3’ | |
| 12 | m *Msi1* forward | 5’-atggatgccttcatgctgggt-3’ | |
|  | m *Msi1* reverse | 5’-ctccgctctacacggaattcg-3’ | |
| 13 | m *Msi2* forward | 5’-atgctgggctaccccaact-3’ | |
|  | m *Msi2* reverse | 5’-cgcgccaaaattcggttga-3’ | |
| 14 | m *Arg1* forward | 5' -gtgaagaacccacggtctgt- 3' | |
|  | m *Arg1* reverse | 5'-ctggttgtcaggggagtgtt-3' | |
| 15 | m *Mrc1* forward | 5'-catgaggcttctcctgcttctg-3' | |
|  | m *Mrc1* reverse | 5'-ttgccgtctgaactgagatgg-3' | |
| 16 | m *Il10* forward | 5'-ggactttaagggttacttgggttgcc-3' | |
|  | m *Il10* reverse | 5'-cattttgatcatcatgtatgcttct-3' | |
| 17 | m *Tgfb* forward | 5'-ccctatatttggagcctgga-3' | |
|  | m *Tgfb* reverse | 5'-gttggttgtagagggcaagg-3' | |
| 18 | m *Ccl17* forward | 5'-tcagatgctgctcctggctg-3' | |
|  | m *Ccl17* reverse | 5'-gcttgcccaggacagtcaga-3' | |
| 19 | m *Ccl2* forward | 5'-taaaaacctggatcggaaccaaa-3' | |
|  | m *Ccl2* reverse | 5'-gcattagcttcagatttacgggt-3' | |
| 20 | m *Nos2* forward | 5'-tgctaatgcgaaaggtcatgg-3' | |
|  | m *Nos2* reverse | 5'-cccaaatgtgcttgtcaccac-3' | |
| 21 | m *Il12* forward | 5'-gacttgaagatgtaccagacag-3' | |
|  | m *Il12* reverse | 5'-gagatgagatgtgatgggag-3' | |
| 22 | m *Tnfa* forward | 5'-agcccacgtcgtagcaaaccaccaa-3' | |
|  | m *Tnfa* reverse | 5'-acacccattcccttcacagagcaat-3' | |
| 23 | m *Il6* forward | 5'-cttcttgggactgatgctggtg-3' | |
|  | m *Il6* reverse | 5'-caggatttcccagagaacatgtg-3' | |
| 24 | m *Il1b* forward | 5’-gaaatgccaccttttgacagtg-3’ | |
|  | m *Il1b* reverse | 5’-tggatgctctcatcaggacag-3’ | |
| 25 | m *Cxcl2* forward | 5'-ccaaccaccaggctacagg-3' | |
|  | m *Cxcl2* reverse | 5'-gcgtcacactcaagctctg-3' | |
| 26 | m *Hes1* forward | 5’-atagctcccggcattccaag-3’ | |
|  | m *Hes1* reverse | 5’-gcgcggtatttccccaaca-3’ | |
|  | | | |
| **For ChIP assays for H3K27me3 and JMJD3 binding** | | | |
| 1 | m *Acsl1* forward | | 5’-ccacactgaccagttctggacac-3’ |
|  | m *Acsl1* reverse | | 5’-caggactgtgagttaccatgtgcg-3’ |
| 2 | m *Adrp* forward | | 5’-gctccttagtgatatcagccaac-3’ |
|  | m *Adrp* reverse | | 5’-ttccctgaacccttatgactcc-3’ |
| 3 | m *Psap* forward | | 5’-gccactacacccggctagaacc-3’ |
|  | m *Psap* reverse | | 5’-ggttctgggggctggagag-3’ |
| 4 | m *Fat* forward | | 5’-gaatcacaggagcatgtaggtctg-3’ |
|  | m *Fat* reverse | | 5’-ctctgatccaataggggcatgtat-3’ |
| 5 | m *Arg1* forward | | 5’-gggaaataaatgatgccttcc-3’ |
|  | m *Arg1* reverse | | 5’-tgagtcaaggcttcaagttctg-3’ |
| 6 | m *Mrc1* forward | | 5’-tttcacttgaaggtaaaccatctg-3’ |
|  | m *Mrc1* reverse | | 5’-ccagacgttcaacaagaattaaga-3’ |
| 7 | m *Il10* forward | | 5’-ctgaagggaaggtccagaca-3’ |
|  | m *Il10* reverse | | 5’-ctgtgctgtcgggtagacct-3’ |
| 8 | m *Tgfb* forward | | 5’-tcgactccatctccaggtgt-3’ |
|  | m *Tgfb* reverse | | 5’-gctagctgctccctccaact-3’ |
| 9 | m *Ccl17* forward | | 5’-aaactggtaagtggccatgc-3’ |
|  | m *Ccl17* reverse | | 5’-gcacctgctaatgaatggtg-3’ |
| 10 | m *Ccl2* forward | | 5’-tatgcctggctcctggtaag-3’ |
|  | m *Ccl2* reverse | | 5’-gagtcgtggagacaatgactg-3’ |
|  | | | |
| **For RNA IP** | | | |
| 1 | m *Spen* forward | 5’-ccgcacatggagaacaggag-3’ | |
|  | m *Spen* reverse | 5’-aaggcttcagcacgagtccc-3’ | |
| 2 | m *Numb* forward | 5’-taggcaggagccctggcttgttt-3’ | |
|  | m *Numb* reverse | 5’-accaggacaacactgcttgctcatc-3’ | |
